# Variant calling on the GRCh38 assembly with the data from phase three of the 1000 Genomes Project

**DOI:** 10.12688/wellcomeopenres.15126.2

**Published:** 2019-12-30

**Authors:** Ernesto Lowy-Gallego, Susan Fairley, Xiangqun Zheng-Bradley, Magali Ruffier, Laura Clarke, Paul Flicek, The 1000 Genomes Project Consortium

**Affiliations:** 1 European Molecular Biology Laboratory, European Bioinformatics Institute, Wellcome Genome Campus, Hinxton, Cambridge, CB10 1SD, UK

**Keywords:** Genomics, population genetics, variant calling, single nucleotide variation, variant discovery

## Abstract

We present a set of biallelic SNVs and INDELs, from 2,548 samples spanning 26 populations from the 1000 Genomes Project, called *de novo* on GRCh38. We believe this will be a useful reference resource for those using GRCh38. It represents an improvement over the “lift-overs” of the 1000 Genomes Project data that have been available to date by encompassing all of the GRCh38 primary assembly autosomes and pseudo-autosomal regions, including novel, medically relevant loci. Here, we describe how the data set was created and benchmark our call set against that produced by the final phase of the 1000 Genomes Project on GRCh37 and the lift-over of that data to GRCh38.

## Introduction

The 1000 Genomes Project produced a deep catalogue of human genomic variation and sequenced more than 2600 samples from 26 different populations. It completed its final phase (“phase three”), with the release of more than 85 million variants of various types and phased haplotypes for those variants ^1^. This data has been widely used by the scientific community for genotype imputation and many other applications ^2^. The strategy adopted by the project consisted of sequencing samples using low coverage whole genome sequencing (WGS) and whole exome sequencing (WES), and the alignment of that sequence data to a version of the GRCh37 human reference genome, which included decoy sequences for optimal read mapping.

While the 1000 Genomes Project was based on GRCh37, the current version of the human reference assembly is GRCh38, which was released by the Genome Reference Consortium (GRC) in 2013. This is the most comprehensive representation of the human genome currently available, as demonstrated by Schneider *et al.*, whose work illustrates the superiority of GRCh38 over GRCh37 ^3^. Specifically GRCh38 is a better basis for annotation, alters read alignment (even in unchanged regions of the genome) and “impacts variant interpretation at clinically relevant loci” ^3^.

To make full use of GRCh38, there has been a need for widely used genomic reference data sets, like the 1000 Genomes Project data, to be made available on the assembly so that pipelines and analyses that rely on such additional reference materials can use GRCh38 and benefit from its improvements.

dbSNP have facilitated the use of the 1000 Genomes Project variation data on GRCh38 by transferring the variant calls to the new assembly using a method relying on an alignment created between GRCh37 and GRCh38. The alignment is then used to determine equivalent locations between the two assemblies, allowing variation data to be “lifted-over”. Files from dbSNP are reformatted into a standard VCF by the European Variation Archive (EVA) and shared as part of our resources through the 1000 Genomes FTP site ^4^ and also via the Ensembl genome browser ^5^.

Lift-over approaches, however, have several limitations. First, in order to be able to transfer a variant from one assembly to another, it is necessary to be able to map between the genomes at the variant’s original location, which is not always possible. In the lift-over process mentioned above there were over 2.3 million VCF records which could not be transferred to the GRCh38 assembly. Second, even when a variant can be lifted-over it does not follow that the underlying read alignments supporting the variant identification on the original assembly would transfer to the identified location in the new assembly. Indeed, alterations to the genome have an impact on read alignments even in unaltered regions of the genome ^3^. Thus, despite a variant being lifted-over, there is no guarantee that it would have been called at the identified location in the new genome as the underlying evidence may vary. Finally, where novel sequence is introduced in GRCh38, it is unlikely that the lift-over approach will be effective as this sequence was not represented in the older assembly and, therefore, not included in the original variation discovery process. Examples of this can be seen where gaps in the assembly have been closed, including at medically relevant loci where gaps have been closed, such as *INPP5D*, *DPP6* and *IKZF1* ^3^, and which are considered below.

To realise the benefits and address the limitations described above, we created new call sets from alignments of the original 1000 Genomes Project read data to GRCh38, initially releasing only biallelic SNVs (described in a previous version of this note) and now updating to biallelic SNVs and INDELs. While this work does not replicate the full repertoire of analyses employed by the 1000 Genomes Consortium, it aims to give a consistent *de novo* call set spanning all of the GRCh38 primary assembly autosomes and pseudo-autosomal regions, and to produce a call set with similar, although not identical, properties to that produced by the 1000 Genomes Project while using a simpler methodology.

To create an updated variation call set from the 1000 Genomes Project data, we adopted a multi-caller approach and used previously described alignments ^6^. With the aim of sharing this data in a timely manner, we adopted an incremental approach to generating and releasing data sets. Initially, we released only biallelic SNVs, which represent the vast majority of the SNVs present in the human genome. Phase three of the 1000 Genomes Project reported that 99.6% of the 81.4 million SNVs they reported are biallelic. Here, we extend our biallelic SNV call set by adding biallelic INDELs. We anticipate future updates to incorporate calls on new populations and the non-pseudo autosomal regions of chromosome X.

## Methods

### Input data

The methods used for sample collection, library construction, and sequencing are described in the previous 1000 Genomes Project publications ^1, 7, 8^. The read data used for this analysis used similar criteria to the final phase of the 1000 Genomes Project. Only Illumina sequence data with reads longer than 70 bp (WGS) and 68 bp (WES) were used. This data was aligned to GRCh38 as previously described ^6^. The complete list of the whole genome and whole exome sequencing alignment files used as the input for generating the callsets can be found on our FTP site at ftp://ftp.1000genomes.ebi.ac.uk/vol1/ftp/data_collections/1000_genomes_project/1000genomes.low_coverage.GRCh38DH.alignment.index and at ftp://ftp.1000genomes.ebi.ac.uk/vol1/ftp/data_collections/1000_genomes_project/1000genomes.exome.GRCh38DH.alignment.index.

### Reference genome

We used the full GRCh38 reference, including ALT contigs, decoy and EBV sequences (accession GCA_000001405). In addition, more than 500 HLA sequences compiled by Heng Li from the IMGT/HLA database provided by the Immuno Polymorphism Database (IPD) ^9^ are included. The reference genome can be accessed at ftp://ftp.1000genomes.ebi.ac.uk/vol1/ftp/technical/reference/GRCh38_reference_genome/.

### Ethical considerations

Information concerning ethical approval and the informed consent procedure for the 1000 Genomes Project can be found at https://www.internationalgenome.org/sites/1000genomes.org/files/docs/Informed%20Consent%20Background%20Document.pdf.

### Quality control of the alignment files

We adopted a similar quality control process to that used in the final phase of the 1000 Genomes Project. The following describes the methods used in their entirety. Chk_indel_rg was applied to discard alignment files with an unbalanced ratio of short insertions and deletions (greater than 5). Picard CollectWgsMetrics was used with the whole genome files and those with mean non-duplicated aligned coverage level ≤2x were discarded. In the case of the exome files, we used Picard CollectHsMetrics using the exome target coordinates at ftp.1000genomes.ebi.ac.uk/vol1/ftp/data_collections/1000_genomes_project/working/20190125_coords_exon_target/ and keeping files where more than 70% of the target regions have 20× or greater coverage. In addition, VerifyBAMID ^10^ was used to assess sample contamination and mix-ups and the following cutoffs were used:

- free_mix > 0.03 and chip_mix > 0.02 for whole genome files

- free_mix > 0.035 and chip_mix > 0.02 for exome files

Only files passing the quality assessment were used in variant calling.

### Variant discovery

Callers were selected in consultation with members of the original 1000 Genomes Project, using their prior knowledge of caller output and the feasibility of running callers on the data set. This enabled us to take advantage of knowledge of a wide range of callers and their performance with this data, the profile of which is now atypical. Specifically, these data are low-coverage from samples representing a diverse range of populations, which necessitates a strategy relying on joint genotyping and the presence of many individuals from a given population. Four supporting call sets were created, using different callers and combinations of the exome and WGS sequence data.

**Figure 1.**
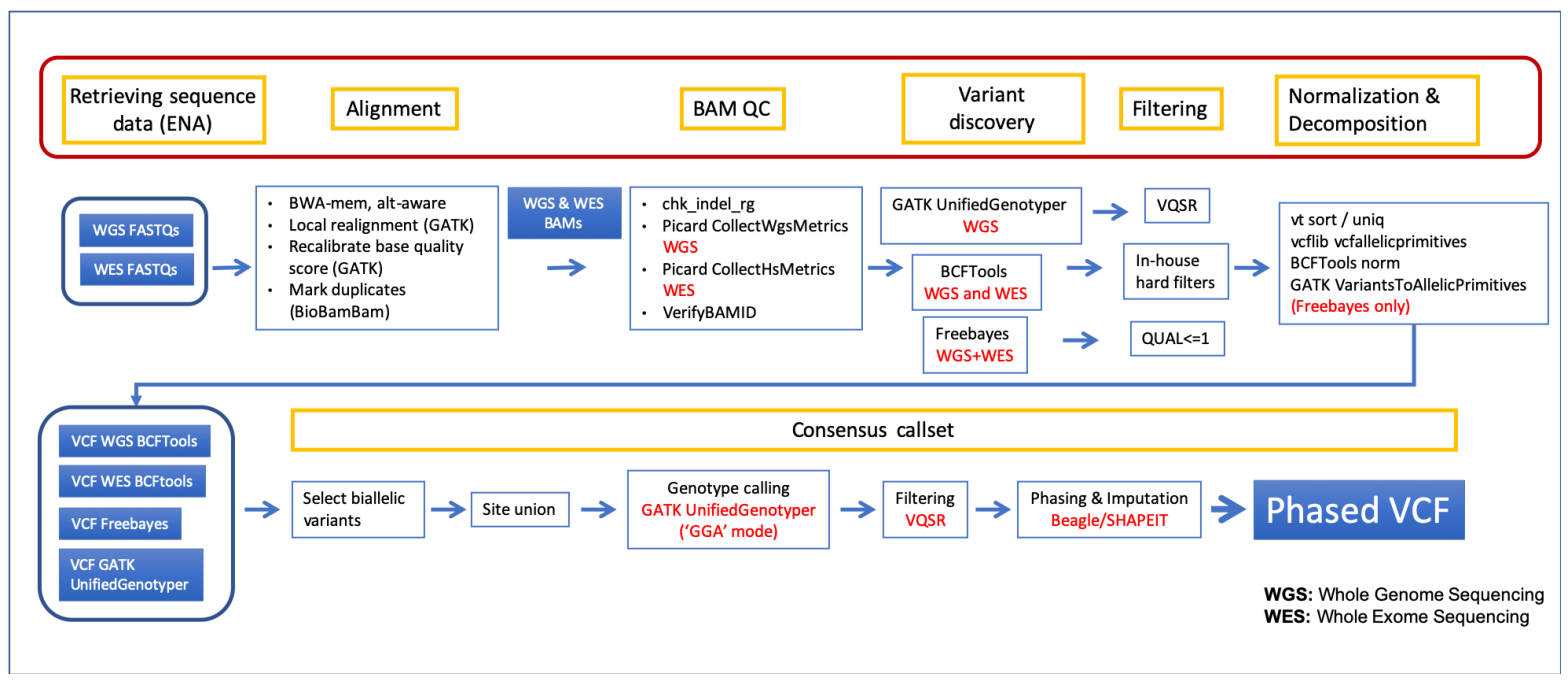
Schematic representation of our approach illustrating the entire process from the alignment files previously generated to the generation of the four supporting callsets and finally to the production of the final phased consensus callset. VCF, variant call format; WGS, whole-genome sequencing; WES, whole-exome sequencing; VQSR, variant quality score recalibration.

A total of 2,659 WGS and 2,498 WES BAMs corresponding to 2,698 samples ^6^ were used for variant identification. Figure 1 details the analysis of the alignment files with three established methods ( BCFtools version 1.3.1-220-g9f38991, Freebayes version v1.0.2-58-g054b257 and GATK UnifiedGenotyper ^11^ version 3.5-0-g36282e4). BCFtools was used to analyse WGS and WES files in two independent runs, GATK UnifiedGenotyper was used only with WGS files and Freebayes was used to analyse everything together (WGS+WES). Calls were made only on the primary assembly autosomes and pseudo-autosomal regions. The following command lines were used for each of the methods to perform joint genotyping:

    • BCFtools with the WGS files:
bcftools mpileup -E -a DP -a SP -a AD -P ILLUMINA \
  -pm3 -F0.2 -C50 -d 700000 \
  -f $ref.fa $file.bam | bcftools call -mv -O z \
  --ploidy GRCh38 -S $samples.ped -o $out.vcf.gz

    • GATK UnifiedGenotyper with the WGS files:
java -Xmx6g -jar GenomeAnalysisTK.jar \
  -T UnifiedGenotyper \
  -R $ref.fa \
  -I $file.bam \ 
  -o $out.vcf.gz \ 
  -dcov 250 \
  -stand_emit_conf 10 \ 
  -glm both \
  --genotyping_mode GENOTYPE_GIVEN_ALLELES \
  --dbsnp ALL_20141222.dbSNP142_human_GRCh38.snps.vcf.gz \
  -stand_call_conf 10
    • BCFtools with the WES files:
bcftools mpileup -E -a DP -a SP -a AD -P ILLUMINA \
  -pm3 -F0.2 -C50 -d 1400000 \
  -f $ref.fa $file.bam | bcftools call -mv -O z \
  --ploidy GRCh38 -S $samples.ped -o $out.vcf.gz

    • Freebayes with the WGS+WES files:
freebayes --genotyping-max-iterations 10 \ 
  --min-alternate-count 3 \
  --max-coverage 2000000 \
  --min-mapping-quality 1 \
  --min-alternate-qsum 50 \
  --min-base-quality 3 \
  -f $ref.fa \
  -b $file.bam | bgzip -c > $out.vcf.gz

### Variant filtering

Our variant discovery pipeline produced four initial call sets as described above. To create an integrated call set, we discarded the variants falling in the centromeres, as these are regions of low complexity that hinder variant calling. Variants on the Y chromosome or in regions of the X chromosome outside the pseudo-autosomal regions were discarded due to the ploidy settings used in this work. Additionally, the initial call sets were filtered using different methods and parameters depending on the call set:

***GATK UnifiedGenotyper call set***. We used the VariantScoreRecalibration (VQSR) ^11^ method following the GATK best practices and GATK training call sets. The combination of commands and parameters we used were different depending on the variant type being analysed. For SNPs we used GATK VariantRecalibrator and ApplyRecalibration as follows:


java -jar GenomeAnalysisTK.jar \
  -T VariantRecalibrator \
  -R $ref.fa \
  -input $file.vcf.gz \ 
  -resource:hapmap,known=false,training=true,truth=true,prior=15.0 hapmap_3.3.hg38.vcf.gz \ 
  -resource:omni,known=false,training=true,truth=true,prior=12.0 1000G_omni2.5.hg38.vcf.gz \ 
  -resource:1000G,known=false,training=true,truth=false,prior=10.0 1000G_phase1.snps.high_confidence.hg38.vcf.gz \
  -resource:dbsnp,known=true,training=false,truth=false,prior=2.0 dbsnp_146.hg38.vcf.gz \ 
  -an DP \
  -an QD \
  -an FS \
  -an SOR \
  -an MQ \
  -an MQRankSum \ 
  -an ReadPosRankSum \ 
  -an InbreedingCoeff \
  -mode SNP \
  -tranche 100.0 -tranche 99.9 -tranche 99.0 -tranche 98.0 -tranche 97.0 -tranche 96.0 -tranche 95.0 -tranche 92.0 -tranche 90.0 -tranche 85.0 -tranche 80.0 -tranche 75.0 -tranche 70.0 -tranche 65.0 -tranche 60.0 -tranche 55.0 -tranche 50.0 \
  -recalFile recalibrate_SNP.recal \
  -tranchesFile recalibrate_SNP.tranches \ 
  -rscriptFile recalibrate_SNP_plots.R


And:


java -jar GenomeAnalysisTK.jar
  -T ApplyRecalibration \
  -R $ref.fa \
  -input $file.vcf.gz \
  -mode SNP \
  --ts_filter_level 99.9 \
  -recalFile recalibrate_SNP.recal \
  -tranchesFile recalibrate_SNP.tranches | bgzip -c > recalibrated_snps_raw_indels.vcf.gz


And for INDELs we used:


java -jar GenomeAnalysisTK.jar \
  -T VariantRecalibrator \
  -R $ref.fa \
  -input recalibrated_snps_raw_indels.vcf.gz \ 
  -resource:mills,known=false,training=true,truth=true,prior=12.0 Mills_and_1000G_gold_standard.indels.hg38.vcf.gz \ 
  -resource:dbsnp,known=true,training=false,truth=false,prior=2.0   dbsnp_146.hg38.vcf.gz \
  -an QD \
  -an DP \
  -an FS \
  -an SOR \
  -an ReadPosRankSum \
  -an MQRankSum \
  -an InbreedingCoeff \
  -mode INDEL \
  -tranche 100.0 -tranche 99.9 -tranche 99.0 -tranche 98.0 -tranche 97.0 -tranche 96.0 -tranche 95.0 -tranche 92.0 -tranche 90.0 -tranche 85.0 -tranche 80.0 -tranche 75.0 -tranche 70.0 -tranche 65.0 -tranche 60.0 -tranche 55.0 -tranche 50.0 \
  -recalFile recalibrate_INDEL.recal \
  -tranchesFile recalibrate_INDEL.tranches \
  -rscriptFile recalibrate_INDEL_plots.R \
  --maxGaussians 4


And:


java -jar GenomeAnalysisTK.jar \
  -T ApplyRecalibration \
  -R $ref.fa \
  -input recalibrated_snps_raw_indels.vcf \
  -mode INDEL \
  --ts_filter_level 80.0 \
  -recalFile recalibrate_INDEL.recal \
  -tranchesFile recalibrate_INDEL.tranches | bgzip -c > recalibrated_variants.vcf.gz


***BCFTools call sets***. Filtering was based on variant annotations. The variant annotations used in the filtering and their respective cutoff values were established by comparing the distribution of the annotation values in the true and false positive sites. In the case of the low coverage data we compared the sites in chromosome 20 only and in the case of the exome data we used sites on all chromosomes. We considered true positives to be the sites identified in our call set for genome NA12878 that were also present in the gold-standard call set generated for the same sample by Genome in a Bottle (GIAB). GIAB’s calls for NA12878 are the result of an effort to integrate data generated by 13 different sequencing technologies and analysis methods ^12^. Sites that were present in our call sets and absent in GIAB were considered false positive sites. Table 1 and Table 2 show the variant annotations and cutoff values used for the SNPs and INDELs with the low coverage data and Table 3 and Table 4 show the annotations and cutoff values used for the exome data with the SNPs and INDELs respectively.

**Table 1.** Variant annotations and cutoff values used for SNPs identified using the low coverage data.

Annotation	Description	Cutoff value
INFO/DP	Raw read depth	>24,304
INFO/MQ	Average mapping quality	<34
INFO/MQ0F	Fraction of MQ0 reads (smaller is better)	>0.049737
INFO/HOB	Bias in the number of HOMs number (smaller is better)	>0.1643732
INFO/SGB	Segregation based metric	>2347.043
INFO/SGB	Segregation based metric	<-64440.286
QUAL	Variant quality	<20

**Table 2.** Variant annotations and cutoff values used for INDELs identified using the low coverage data.

Annotation	Description	Cutoff value
INFO/DP	Raw read depth	>23,758
INFO/MQ	Average mapping quality	<41
INFO/MQ0F	Fraction of MQ0 reads (smaller is better)	>0.009913696
INFO/HOB	Bias in the number of HOMs number (smaller is better)	>0.20265508
INFO/SGB	Segregation based metric	>2143.8876
INFO/SGB	Segregation based metric	<-29513.557
INFO/IDV	Maximum number of reads supporting an indel	>51
INFO/IMF	Maximum fraction of reads supporting an indel	<0.387097
QUAL	Variant quality	<20

**Table 3.** Variant annotations and cutoff values used for SNPs identified using the exome data.

Annotation	Description	Cutoff value
INFO/DP	Raw read depth	>656,519
INFO/MQ	Average mapping quality	<38
INFO/MQ0F	Fraction of MQ0 reads (smaller is better)	>0.0146629
INFO/HOB	Bias in the number of HOMs number (smaller is better)	>0.1536016
INFO/SGB	Segregation based metric	>57489.21
INFO/SGB	Segregation based metric	<-226326.93
QUAL	Variant quality	<20

**Table 4.** Variant annotations and cutoff values used for INDELs identified using the exome data.

Annotation	Description	Cutoff value
INFO/MQ	Average mapping quality	<45
INFO/MQ0F	Fraction of MQ0 reads (smaller is better)	>0.002034686
INFO/HOB	Bias in the number of HOMs number (smaller is better)	>0.269603
INFO/SGB	Segregation based metric	>53165.5
INFO/SGB	Segregation based metric	<-85919.729
INFO/IMF	Maximum fraction of reads supporting an indel	<0.3323922
QUAL	Variant quality	<20

These cutoff values were applied using the following command:

     • SNPs from the low coverage data:


bcftools filter -s GIABFILTER \ 
  -e'INFO/DP>24304 | MQ<34 | MQ0F>0.049737 | HOB>0.1643732 | SGB>2347.043 | SGB<-64440.286 | QUAL<20' \
  $file.snps.vcf.gz \
  -o $out.snps.filtered.vcf.gz -O z


     • INDELs from the low coverage data:


bcftools filter -s GIABFILTER \
  -e'INFO/DP>23758 | MQ<41 | MQ0F>0.009913696 | HOB>0.20265508 | SGB>2143.8876 | SGB<-29513.557 | IDV>51 | IMF<0.387097 | QUAL<20' $file.indels.vcf.gz -o $out.indels.filtered.vcf.gz -O z


     • SNPs from the exome data:


bcftools filter -sGIABFILTER \
  -e'INFO/DP>656519 | MQ<38 | MQ0F> 0.0146629| HOB>0.1536016 | SGB>57489.21 | SGB < -226326.93| QUAL<20' $file.snps.vcf.gz \
  -o  $out.snps.filtered.vcf.gz -O z


     • INDELs from the exome data:


bcftools filter -sGIABFILTER \
  -e'MQ<45 | MQ0F>0.002034686| HOB> 0.269603| SGB>53165.5 | SGB<-85919.729 | IMF<0.3323922 | QUAL<20' $file.indels.vcf.gz \
  -o $out.indels.filtered.vcf.gz -O z


***Freebayes call set***. We used a simple hard filter that discarded variants having a QUAL value less than or equal to 1 since this cutoff value has been recommended by the author of Freebayes (personal communication, [Erik Garrison]) and proved to be effective in filtering variants in phase three of the 1000 Genomes Project. This filter was applied using the following command:


bcftools filter -sQUALFILTER -e'QUAL<1' $file.vcf.gz \ 
  -o $file.filtered.vcf.gz -O z


### Generating consensus call sets

***Biallelic SNVs***. First, each call set was normalized using the following combination of tools:


bcftools norm -f ref.fa -o norm.vcf.gz -m '-both’ in.vcf.gz -Oz


in order to normalize and left-align INDELs and to split the multiallelic sites into multiple rows. Then we run:


vcfallelicprimitives norm.vcf.gz --keep-info --keep-geno | vt sort - | vt uniq - | bgzip -c > norm.aprimitives.vcf.gz


where vcflib vcfallelicprimitives (version v1.0.0-rc1) was used to decompose the complex variants and vt ^13^ (version 0.5) was used to sort and unify resulting variants. After this we run:


bcftools norm -f ref.fa -o norm.aprimitives.merged.vcf.gz -m '+both’ norm.aprimitives.vcf.gz -Oz


this merges the multiallelic sites into single rows. Finally, the multiallelic sites were discarded in the following step:


bcftools view -o norm.aprimitives.merged.biallelic.vcf.gz -O z -m2 -M2 norm.aprimitives.merged.vcf.gz


This normalization procedure is necessary as different variant callers may describe the same variant in different ways, which makes comparison difficult and affects the integration of the call sets. Additionally, GATK VariantsToAllelicPrimitives was used to decompose the multi-nucleotide polymorphisms (MNPs) that were present in the Freebayes call set.

Finally, we generated a consensus call set by taking the union of the biallelic sites from each call set and calculating the genotype likelihoods for each site using GATK UnifiedGenotyper in ‘genotype_given_alleles’ (GGA) mode using the following command line:


java -jar GenomeAnalysisTK.jar \
  -T UnifiedGenotyper \
  -R $ref.fa \
  -I input.$chr:$start-$end.bam \
  -glm SNP \
  --intervals $chr:$start-$end \
  --intervals integrated.biallelic.sites.vcf.gz \
  --output_mode EMIT_ALL_SITES \
  --alleles integrated.biallelic.sites.vcf.gz \
  --interval_set_rule INTERSECTION \
  --genotyping_mode GENOTYPE_GIVEN_ALLELES \
  --max_deletion_fraction 1.5


Where $chr:$start-$end is the genomic chunk that is being analysed and integrated.biallelic.sites.vcf.gz is the VCF containing the union of the biallelic sites for which the genotype likelihoods will be calculated.

We then filtered the variants using Variant Quality Score Recalibration (VQSR) with the same parameters and training call sets that were described above and used for filtering the supporting call set generated using GATK UnifiedGenotyper. GATK ApplyRecalibrator was used with a --ts_filter_level value of 99.5, chosen to balance sensitivity and specificity. This gave a consensus biallelic SNV call set, used as the basis of the initial biallelic SNV-only call set.

***Biallelic SNVs and INDELs***. To add the INDEL variants to the SNV-only data set (for our second data release), we extracted the INDELs from the initial BCFTools, GATK and Freebayes call sets described above and generated a consensus call set by taking the union of the normalized biallelic INDELs from each call set. Then, we calculated the genotype likelihoods for each site using again GATK UnifiedGenotyper in ‘genotype_given_alleles’ (GGA) mode using the following command line:


java -jar GenomeAnalysisTK.jar \
  -T UnifiedGenotyper \
  -R $ref.fa \
  -I input.$chr:$start-$end.bam \
  -glm INDEL \
  --intervals $chr:$start-$end \
  --intervals integrated.biallelic.indel.sites.vcf.gz \
  --output_mode EMIT_ALL_SITES \
  --alleles integrated.biallelic.indel.sites.vcf.gz \
  --interval_set_rule INTERSECTION \
  --genotyping_mode GENOTYPE_GIVEN_ALLELES \
  --max_deletion_fraction 1.5



Where $chr:$start-$end is the genomic chunk that is being analysed and integrated.biallelic.indel.sites.vcf.gz is the VCF containing the union of the biallelic INDEL sites for which the genotype likelihoods will be calculated.

The next step consisted of filtering this INDEL-only consensus call set, and as we did for the SNV-only call set, we used the GATK Variant Quality Score Recalibration (VQSR) method, this time running ApplyRecalibrator with a --ts_filter_level value of 99.0. This is lower than the value of 99.5 used with the SNV-only data set. This was chosen to focus on specificity, due to the greater challenges in INDEL calling, while also balancing sensitivity.

Finally, the INDEL-only consensus call set was merged to the initial SNV-only call set by using bcftools concat.

### Phasing and imputation of the consensus call set

***Biallelic SNVs***. The VCF file containing the genotype likelihoods obtained following the procedure described above was divided into single chromosome VCF files that were further divided into genomic chunks containing 2,100 sites of which 600 were shared between consecutive chunks. These chunks were processed in parallel by Beagle ^14^ by using the following command:


java -jar beagle.08Jun17.d8b.jar \
  chrom=$chr:$start-$end \
  gl=$chr.biallelic.GL.vcf.gz \
  out=$chr.$start.$end.beagle \
  niterations=15


Where $chr.biallelic.GL.vcf.gz is the VCF file containing the genotype likelihoods.

After processing all the chunks with Beagle, the initial set of genotypes and haplotypes were phased using SHAPEIT2 ^15^ (version v2.r837) onto a highly accurate haplotype scaffold also created by SHAPEIT2 using microarray genotype data available on the same samples. This scaffold was obtained by leveraging family information and running SHAPEIT2 in two different independent runs on either the Illumina Omni 2.5 or Affymetrix 6.0 microarray data that was generated as part of the 1000 Genomes Project. To create the microarray scaffolds SHAPEIT2 was run using the following settings (--window 0.5, --states 200, --burn 10, --prune 10, --main 50, --duohmm) and SNPs with a missing data rate above 10% and a Mendel error rate above 5% were removed before phasing. Genotypes called by Beagle with a posterior probability greater than 0.995 were fixed as known genotypes and haplotypes estimated by Beagle were used to initialize the SHAPEIT2 phasing. This phasing was run in chunks of 12,250 sites with 3,500 sites overlapping between consecutive chunks. When phasing the calls derived from sequence data onto the microarray scaffolds SHAPEIT2 was run using the following command:


shapeit -call \
  --input-gen input.shapeit.$chr.gen.gz input.shapeit.$chr.gen.sample \
  --input-init input.shapeit.$chr.hap.gz input.shapeit.hap.sample \
  --input-scaffold chip.omni.snps.$chr.haps chip.omni.snps.$chr.sample chip.affy.snps.$chr.haps chip.affy.snps.$chr.sample  \
  --input-map $chr.gmap.gz \
  --input-thr 1 \
  --window 0.1 \
  --states 400 \
  --states-random 200 \
  --burn 0 \
  --run 12 \
  --prune 4 \
  --main 20 \
  --input-from $chunk_start \
  --input-to $chunk_end  \
  --output-max out.$chr.$chunk_start.$chunk_end.haps.gz out.$chr.$chunk_start.$chunk_end.haps.sample


Where --input-gen specifies the genotype/GL input data from Beagle, --input-init specifies the haplotypes from Beagle, --input-map specifies the genetic map used in the estimation, --input-scaffold gives the SNP-array derived haplotype scaffold obtained from SHAPEIT2. The genetic map used was downloaded from https://data.broadinstitute.org/alkesgroup/Eagle/downloads/tables/genetic_map_hg38_withX.txt.gz. Each of the phased chunks resulting from running SHAPEIT2 were joined together using the program ligateHAPLOTYPES.

The strategy described here was used in the final phase of the 1000 Genomes Project and has been shown to produce low error rates for genotype calls ^16^.

The pipelines used in this work were implemented using the eHive workflow system ^17^ and modules developed in Perl and Python, which have been packaged for ease of deployment. All the analyses were run in parallel on a high-throughput compute cluster to ensure completion in a reasonable timeframe. Code is publicly available via GitHub (see software availability section) ^17– 19^.

***Biallelic SNVs and INDELs***. The merged VCF for biallelic SNVs and INDELs was phased and imputed using Beagle/SHAPEIT2, using the same process described above for phasing and imputation of the SNV-only data set.

**Figure 2.**
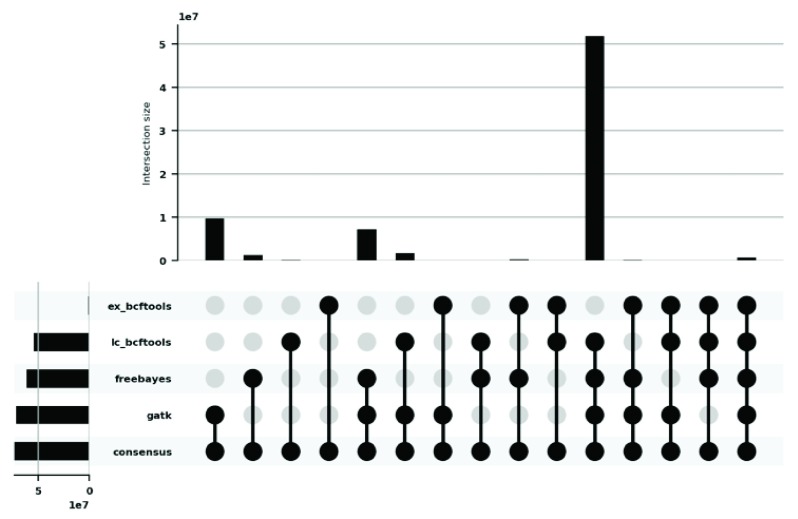
UpSet plot analysing the contribution of each of the four supporting call sets to the final SNV consensus call set. *‘ex_bcftools’* is the call set generated using BCFTools with the WES (Whole exome sequencing) data. *‘lc_bcftools’* is the call set generated using BCFTools with the low coverage WGS (whole genome sequencing) data. *‘freebayes’* is the call set generated using Freebayes with the low coverage WGS+WES data. *‘gatk’* is the call set generated using GATK UnifiedGenotyper with the low coverage WGS data. *‘consensus’* is the final SNV call set generated after integrating the supporting call sets. Vertical bars show the size of the intersection between the call sets. Horizontal bars show the aggregated size of each call set. We used the filtered supporting call sets to generate this plot.

**Figure 3.**
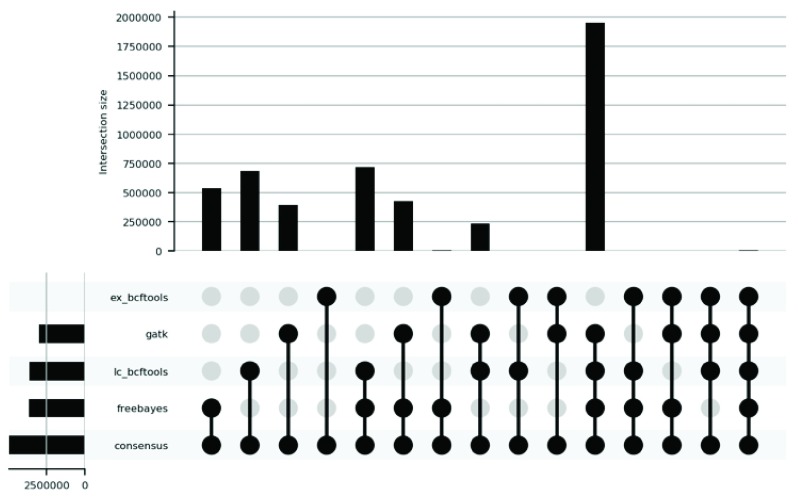
UpSet plot analysing the contribution of each of the four supporting call sets to the final INDEL consensus call set. *‘ex_bcftools’* is the call set generated using BCFTools with the WES (Whole exome sequencing) data. *‘lc_bcftools’* is the call set generated using BCFTools with the low coverage WGS (whole genome sequencing) data. *‘freebayes’* is the call set generated using Freebayes with the low coverage WGS+WES data. *‘gatk’* is the call set generated using GATK UnifiedGenotyper with the low coverage WGS data. *‘consensus’* is the final INDEL call set generated after integrating the supporting call sets. Vertical bars show the size of the intersection between the call sets. Horizontal bars show the aggregated size of each call set. We used the filtered supporting call sets to generate this plot.

To illustrate the contribution of each of the four filtered supporting call sets to the final consensus call set, we generated the plots in Figure 2 and Figure 3, for SNVs and INDELs respectively. Figure 2 shows that the call set that has contributed the most to the final SNV consensus call set is the GATK UnifiedGenotyper call set (71,353,714 variants) followed by the Freebayes call set (61,625,466 variants). In the case of INDELs the call set that has contributed the most to the final INDEL consensus call set is the Freebayes call set (3,649,204 variants) followed by the BCFTools call set used on the low-coverage WGS data (3,602,996 variants).

***Switch error rate of the NA12878 sample***. In order to assess the phasing accuracy in our SNV and INDEL call set we estimated the switch error (SE) rate, which measures the rate for which the phase of a certain haplotype block is incorrectly predicted in the comparison with a true haplotype block. For example, if the correct haplotype is 000111|111000 and the predicted haplotype is 00000|111111, then we count one switch error between positions 3 and 4. In order to estimate these kind of errors we have used WhatsHap ‘compare’ (version 0.18) ^20^ with our phased variants for sample NA12878 and using the GIAB call set for this same sample as the gold-standard phased reference. The SE has been calculated for each autosome by using the following command for SNVs:


whatshap compare --sample NA12878 \
     --only-snvs NA12878.phased.GIAB.snps.chr${i}.vcf.gz \ 
     combined.NA12878.phased.query.snps.chr${i}.vcf.gz


And for INDELs:


whatshap compare --sample NA12878 \
     NA12878.phased.GIAB.indels.chr${i}.vcf.gz \ 
     combined.NA12878.phased.query.indels.chr${i}.vcf.gz


We estimated the SE rates resulting from the following comparisons:

1. our extended call set with GIAB on GRCh38
2. lift-over call set with GIAB on GRCh38
3. P3 call set with GIAB on GRCh37

And the results of these comparisons can be seen in Table 5 for SNVs and Table 6 for INDELs, where we can see that the average SE rate for SNVs across all the autosomes is lower in our call set (0.71%) than in the lift-over and P3 call sets (0.91% and 1.54% respectively) and is also lower for INDELs (1.78% versus 3.16% and 5.18% respectively).

**Table 5.** Switch error (SE) rates for phased SNVs for NA12878. *‘This_work’* contains the rates for the comparison between our call set and GIAB. *‘lift-over’* contains the rates for the lift-over call set compared to GIAB. *‘P3’* column contains the rates for the phase three call set compared to GIAB.

chromosome	This_work	lift-over	P3
**1**	0.99%	1.15%	2.42%
**2**	0.53%	0.70%	0.70%
**3**	0.48%	0.77%	0.72%
**4**	0.50%	0.66%	2.45%
**5**	0.51%	0.68%	2.30%
**6**	1.40%	1.59%	2.35%
**7**	0.74%	0.93%	1.23%
**8**	0.61%	0.85%	2.16%
**9**	0.43%	0.70%	2.36%
**10**	0.89%	1.17%	1.49%
**11**	0.57%	0.65%	2.50%
**12**	0.44%	0.63%	0.65%
**13**	0.44%	0.64%	0.66%
**14**	0.44%	0.69%	0.68%
**15**	0.61%	0.77%	0.77%
**16**	1.17%	1.35%	0.60%
**17**	1.62%	1.77%	0.82%
**18**	0.53%	0.74%	2.38%
**19**	0.37%	0.67%	2.94%
**20**	0.46%	0.66%	0.59%
**21**	0.44%	0.58%	0.57%
**22**	1.52%	1.73%	2.53%
**AVG**	**0.71%**	**0.91%**	**1.54%**

**Table 6.** Switch error (SE) rates for phased INDELs from NA12878. *‘This_work’* contains the rates for the comparison between our call set and GIAB. *‘lift-over’* contains the rates for the lift-over call set compared to GIAB. *‘P3’* column contains the rates for the phase three call set compared to GIAB.

chromosome	This_work	lift-over	P3
**1**	2.55%	4.85%	9.32%
**2**	0.66%	1.29%	1.32%
**3**	0.44%	1.25%	1.23%
**4**	0.56%	0.93%	7.64%
**5**	0.57%	1.16%	8.35%
**6**	4.67%	7.10%	8.22%
**7**	2.26%	3.26%	3.68%
**8**	0.95%	2.34%	8.70%
**9**	0.40%	1.51%	8.67%
**10**	2.91%	4.55%	5.17%
**11**	0.71%	0.99%	8.68%
**12**	0.43%	1.12%	1.26%
**13**	0.52%	0.89%	0.73%
**14**	0.51%	1.18%	1.13%
**15**	0.58%	1.12%	1.17%
**16**	5.56%	9.14%	1.10%
**17**	6.35%	10.74%	1.53%
**18**	0.56%	1.24%	8.97%
**19**	0.62%	2.01%	12.75%
**20**	0.86%	1.65%	1.47%
**21**	0.73%	1.15%	1.24%
**22**	5.83%	10.04%	11.57%
**AVG**	**1.78%**	**3.16%**	**5.18%**

### Comparison with the Genome in a bottle (GIAB) call set for NA12878

***Biallelic SNVs***. To assess our biallelic SNV call set and compare it to the final phase of the 1000 Genomes Project, we utilised resources from GIAB. Our strategy compares our GRCh38 calls for NA12878 with the NA12878 calls on GRCh38 from GIAB. In addition, we compared the 1000 Genomes calls for NA12878 to those from GIAB on GRCh37. NA12878 was used in benchmarking as GIAB provides an independent gold-standard data set. For other samples in the 1000 Genomes Project panel, such data is not available, making meaningful benchmarking with other samples impossible. The use of a joint genotyping approach precludes applying our method to a single sample where high quality data is available but a population with low coverage and exome data is not. This limits suitable benchmarks to NA12878 and it should be noted that GIAB’s analysis uses only the primary assembly in alignment, giving a different base from which to make calls, which may include reads that would otherwise have aligned to alt sequences. Within these limitations, this approach enables us to benchmark the performance with an independently produced gold-standard and allows us to apply the equivalent benchmark to data from the 1000 Genomes Project on GRCh37, indicating how our call set compares to that produced by the 1000 Genomes Project.

For NA12878, there are no indications that it is an outlier in the 1000 Genomes Project sequence data. It has similar coverage to other samples at 4.6x compared to an average of 6.2x (standard deviation 2.3) for the WGS and 144.1x relative to an average of 84.9x (standard deviation 34.1) for the exome data, and the same technologies are applied across the data set. Given the prevalence of NA12878 data, we would expect the callers to perform well with this sample but, as the NA12878 data in our work is not exceptional in the data set, we believe the results seen in benchmarking NA12878 are likely to broadly reflect performance across the data set.

We used the NA12878 variants from the multi-sample phased SNV-only VCF and compared them with the GIAB sites on GRCh38 downloaded from [ ftp://ftp-trace.ncbi.nlm.nih.gov/giab/ftp/release/NA12878_HG001/latest] (version 3.3.2). For GRCh37 we compared variants from the final phase of the 1000 Genomes Project (downloaded here) with the GRCh37 GIAB variants obtained here (version 3.3.2). Our comparison is restricted to the regions in the autosomes and in the PAR region of chromosome X where GIAB considers calls to be high confidence (on average 77.9%, standard deviation 12.1%, of the bases for each of the chromosomes are in high confidence regions) and was performed using the Nextflow ^21^ workflow accessible from the link in the software availability section.

The result of our comparison is shown in Table 7. The average percentage of sites among all the chromosomes identified in our work that were also present in GIAB represents 96.4% of the total GIAB sites. This percentage is comparable to 97.9% resulting from the comparison with the final phase of the 1000 Genomes Project (phase three - P3). Additionally, the percentage of sites identified in our call set but not in GIAB is 0.5%, which is comparable to the 0.4% obtained in the comparison with 1000 Genomes phase three.

**Table 7.** Site comparison for NA12878 between our call set and Genome in a Bottle (GIAB)-mapped to GRCh38 and between the 1000 Genomes Project phase three (P3) call set and GIAB mapped to GRCh37. Results are shown for each chromosome. *‘Shared (TP)’* are the true positive variants identified in the compared call sets. *‘giab_only (FN)’* are the false negative variants identified by GIAB only. *‘Thiswork_only (FP)’* are the false positive variants identified in our call set only.

Dataset	Shared (TP)	%shared (TP)	giab_only (FN)	%giab_only (FN)	Thiswork_only (FP)	%Thiswork_only (FP)	Total (GIAB)	Total thiswork_only
**Chr1** **(b38)**	238,323	96.37	8,965	3.63	1,347	0.56	247,288	239,670
**Chr1** **(b37)**	242,331	98.09	4,707	1.91	1,700	0.70	247,038	244,031
**Chr2** **(b38)**	237,017	96.42	8,791	3.58	1,264	0.53	245,808	238,281
**Chr2** **(b37)**	260,921	98.14	4,942	1.86	1,209	0.46	265,863	262,130
**Chr3** **(b38)**	214,201	96.17	8,520	3.83	1,134	0.53	222,721	215,335
**Chr3** **(b37)**	218,474	97.93	4,608	2.07	926	0.42	223,082	219,400
**Chr4** **(b38)**	188,608	96.00	7,860	4.00	847	0.45	196,468	189,455
**Chr4** **(b37)**	232,888	97.93	4,927	2.07	888	0.38	237,815	233,776
**Chr5** **(b38)**	181,015	96.26	7,031	3.74	865	0.48	188,046	181,880
**Chr5** **(b37)**	193,359	95.48	9,162	4.52	766	0.39	202,521	194,125
**Chr6** **(b38)**	197,830	96.04	8,151	3.96	940	0.47	205,981	198,770
**Chr6** **(b37)**	191,018	98.05	3,801	1.95	844	0.44	194,819	191,862
**Chr7** **(b38)**	166,888	96.54	5,982	3.46	854	0.51	172,870	167,742
**Chr7** **(b37)**	167,924	97.98	3,464	2.02	712	0.42	171,388	168,636
**Chr8** **(b38)**	145,748	96.24	5,700	3.76	678	0.46	151,448	146,426
**Chr8** **(b37)**	171,950	97.76	3,937	2.24	715	0.41	175,887	172,665
**Chr9** **(b38)**	131,987	96.42	4,899	3.58	635	0.48	136,886	132,622
**Chr9** **(b37)**	132,596	97.84	2,924	2.16	581	0.44	135,520	133,177
**Chr10** **(b38)**	153,504	96.55	5,480	3.45	815	0.53	158,984	154,319
**Chr10** **(b37)**	153,080	97.87	3,338	2.13	648	0.42	156,418	153,728
**Chr11** **(b38)**	154,516	95.83	6,720	4.17	775	0.50	161,236	155,291
**Chr11** **(b37)**	155,511	97.86	3,407	2.14	609	0.39	158,918	156,120
**Chr12** **(b38)**	136,457	96.46	5,008	3.54	745	0.54	141,465	137,202
**Chr12** **(b37)**	148,026	98.03	2,972	1.97	676	0.45	150,998	148,702
**Chr13** **(b38)**	121,294	96.89	3,889	3.11	560	0.46	125,183	121,854
**Chr13** **(b37)**	122,424	98.08	2,395	1.92	423	0.34	124,819	122,847
**Chr14** **(b38)**	99,613	96.03	4,122	3.97	493	0.49	103,735	100,106
**Chr14** **(b37)**	99,543	97.74	2,300	2.26	434	0.43	101,843	99,977
**Chr15** **(b38)**	85,881	96.59	3,031	3.41	386	0.45	88,912	86,267
**Chr15** **(b37)**	87,224	97.95	1,822	2.05	390	0.45	89,046	87,614
**Chr16** **(b38)**	54,542	96.72	1,850	3.28	282	0.51	56,392	54,824
**Chr16** **(b37)**	92,735	97.92	1,967	2.08	424	0.46	94,702	93,159
**Chr17** **(b38)**	73,765	96.69	2,524	3.31	484	0.65	76,289	74,249
**Chr17** **(b37)**	76,187	98.27	1,341	1.73	441	0.58	77,528	76,628
**Chr18** **(b38)**	73,419	96.89	2,360	3.11	344	0.47	75,779	73,763
**Chr18** **(b37)**	93,004	97.97	1,923	2.03	365	0.39	94,927	93,369
**Chr19** **(b38)**	56,210	95.27	2,788	4.73	461	0.81	58,998	56,671
**Chr19** **(b37)**	59,138	97.93	1,248	2.07	376	0.63	60,386	59,514
**Chr20** **(b38)**	64,786	96.78	2,154	3.22	419	0.64	66,940	65,205
**Ch20** **(b37)**	64,827	97.89	1,400	2.11	275	0.42	66,227	65,102
**Chr21** **(b38)**	42,453	96.96	1,329	3.04	225	0.53	43,782	42,678
**Chr21** **(b37)**	43,941	98.13	836	1.87	178	0.40	44,777	44,119
**Chr22** **(b38)**	33,351	96.81	1,099	3.19	193	0.58	34,450	33,544
**Chr22** **(b37)**	36,132	98.16	678	1.84	207	0.57	36,810	36,339
**ChrX** (b38) *	109	93.97	7	6.03	2	1.80	116	111
AVG ** **(b38)**	**129,609**	**96.41**	**4,921**	**3.59**	**670**	**0.53**	**134,530**	**130,280**
**AVG** **(b37)**	**138,329**	**97.86**	**3,095**	**2.14**	**627**	**0.45**	**141,424**	**138,955**

* Only PAR regions ** Not considering chrX for the calculation

***Biallelic SNVs and INDELs***. We also compared the extended call set containing SNVs and INDELs with the GIAB NA12878 call set in the same way that we did for our previous SNV-only call set (see above).

Additionally, we included the 1000 Genomes Project variants lifted to GRCh38 by dbSNP in the comparison with the GRCh38 GIAB sites. The lift-over call set used in this comparison is accessible from [ftp:// ftp.1000genomes.ebi.ac.uk/vol1/ftp/release/20130502/supporting/GRCh38_positions]. The result of this comparison between our call set and the lift-over callset with GIAB on GRCh38 and between P3 and GIAB on GRCh37 can be seen in Table 8 for the SNV sites and Table 9 for the INDEL sites. Our integrated call set contains 96.4% of the total GIAB SNV sites. This percentage is similar to 97.9% resulting from the comparison with P3 and to 97.0% for the comparison between the lift-over and GIAB. Additionally, 0.5% of the SNV sites we identified were not in GIAB, similar to the 0.4% for P3 and to the 0.5% for the lift-over.

**Table 8.** SNV-only site comparison for NA12878 between our call set and Genome in a Bottle (GIAB)-mapped to GRCh38, between the lift-over (chr*_L rows in the table) call set-mapped to GRCh38 and between the 1000 Genomes Project phase 3 (P3) call set and GIAB mapped to GRCh37. Results are shown for each chromosome. ‘ *Shared (TP)*’ are the true positive variants identified in the compared call sets. ‘ *giab_only (FN)*’ are the false negative variants identified by GIAB only. ‘ *Thiswork_only (FP)*’ are the false positive variants identified in our call set only.

Dataset	Shared (TP)	%shared (TP)	giab_only (FN)	%giab_only (FN)	Thiswork_only (FP)	%Thiswork_only (FP)	Total (GIAB)	Total thiswork_only
**Chr1** **(b38)**	238,340	96.38	8,948	3.62	1,270	0.53	**247,288**	**239,610**
**Chr1_L** **(b38)**	241,396	97.62	5,892	2.38	1,799	0.74	**247,288**	**243,195**
**Chr1** **(b37)**	242,331	98.09	4,707	1.91	1,700	0.7	**247,038**	**244,031**
**Chr2** **(b38)**	237,055	96.44	8,753	3.56	1,208	0.51	**245,808**	**238,263**
**Chr2_L** **(b38)**	240,944	98.02	4,864	1.98	1,232	0.51	**245,808**	**242,176**
**Chr2** **(b37)**	260,921	98.14	4,942	1.86	1,209	0.46	**265,863**	**262,130**
**Chr3** **(b38)**	214,315	96.23	8,406	3.77	1,027	0.48	**222,721**	**215,342**
**Chr3_L** **(b38)**	217,446	97.63	5,275	2.37	1,061	0.49	**222,721**	**218,507**
**Chr3** **(b37)**	218,474	97.93	4,608	2.07	926	0.42	**223,082**	**219,400**
**Chr4** **(b38)**	188,516	95.95	7,952	4.05	873	0.46	**196,468**	**189,389**
**Chr4_L** **(b38)**	192,186	97.82	4,282	2.18	761	0.39	**196,468**	**192,947**
**Chr4** **(b37)**	232,888	97.93	4,927	2.07	888	0.38	**237,815**	**233,776**
**Chr5** **(b38)**	180,892	96.2	7,154	3.8	903	0.5	**188,046**	**181,795**
**Chr5_L** **(b38)**	179,468	95.44	8,578	4.56	756	0.42	**188,046**	**180,224**
**Chr5** **(b37)**	193,359	95.48	9,162	4.52	766	0.39	**202,521**	**194,125**
**Chr6** **(b38)**	197,693	95.98	8,288	4.02	1,013	0.51	**205,981**	**198,706**
**Chr6_L** **(b38)**	199,172	96.69	6,809	3.31	1,150	0.57	**205,981**	**200,322**
**Chr6** **(b37)**	191,018	98.05	3,801	1.95	844	0.44	**194,819**	**191,862**
**Chr7** **(b38)**	166,777	96.48	6,093	3.52	895	0.53	**172,870**	**167,672**
**Chr7_L** **(b38)**	168,159	97.27	4,711	2.73	793	0.47	**172,870**	**168,952**
**Chr7** **(b37)**	167,924	97.98	3,464	2.02	712	0.42	**171,388**	**168,636**
**Chr8** **(b38)**	145,659	96.18	5,789	3.82	719	0.49	**151,448**	**146,378**
**Chr8_L** **(b38)**	147,895	97.65	3,553	2.35	665	0.45	**151,448**	**148,560**
**Chr8** **(b37)**	171,950	97.76	3,937	2.24	715	0.41	**175,887**	**172,665**
**Chr9** **(b38)**	131,911	96.37	4,975	3.63	678	0.51	**136,886**	**132,589**
**Chr9_L** **(b38)**	133,365	97.43	3,521	2.57	614	0.46	**136,886**	**133,979**
**Chr9** **(b37)**	132,596	97.84	2,924	2.16	581	0.44	**135,520**	**133,177**
**Chr10** **(b38)**	153,422	96.5	5,562	3.5	853	0.55	**158,984**	**154,275**
**Chr10_L** **(b38)**	153,010	96.24	5,974	3.76	699	0.45	**158,984**	**153,709**
**Chr10** **(b37)**	153,080	97.87	3,338	2.13	648	0.42	**156,418**	**153,728**
**Chr11** **(b38)**	154,414	95.77	6,822	4.23	808	0.52	**161,236**	**155,222**
**Chr11_L** **(b38)**	156,330	96.96	4,906	3.04	717	0.46	**161,236**	**157,047**
**Chr11** **(b37)**	155,511	97.86	3,407	2.14	609	0.39	**158,918**	**156,120**
**Chr12** **(b38)**	136,392	96.41	5,073	3.59	771	0.56	**141,465**	**137,163**
**Chr12_L** **(b38)**	135,131	95.52	6,334	4.48	646	0.48	**141,465**	**135,777**
**Chr12** **(b37)**	148,026	98.03	2,972	1.97	676	0.45	**150,998**	**148,702**
**Chr13** **(b38)**	121,218	96.83	3,965	3.17	588	0.48	**125,183**	**121,806**
**Chr13_L** **(b38)**	122,714	98.03	2,469	1.97	475	0.39	**125,183**	**123,189**
**Chr13** **(b37)**	122,424	98.08	2,395	1.92	423	0.34	**124,819**	**122,847**
**Chr14** **(b38)**	99,551	95.97	4,184	4.03	501	0.5	**103,735**	**100,052**
**Chr14_L** **(b38)**	99,210	95.64	4,525	4.36	501	0.5	**103,735**	**99,711**
**Chr14** **(b37)**	99,543	97.74	2,300	2.26	434	0.43	**101,843**	**99,977**
**Chr15** **(b38)**	85,827	96.53	3,085	3.47	421	0.49	**88,912**	**86,248**
**Chr15_L** **(b38)**	86,887	97.72	2,025	2.28	426	0.49	**88,912**	**87,313**
**Chr15** **(b37)**	87,224	97.95	1,822	2.05	390	0.45	**89,046**	**87,614**
**Chr16** **(b38)**	54,517	96.68	1,875	3.32	285	0.52	**56,392**	**54,802**
**Chr16_L** **(b38)**	55,233	97.94	1,159	2.06	264	0.48	**56,392**	**55,497**
**Chr16** **(b37)**	92,735	97.92	1,967	2.08	424	0.46	**94,702**	**93,159**
**Chr17** **(b38)**	73,701	96.61	2,588	3.39	502	0.68	**76,289**	**74,203**
**Chr17_L** **(b38)**	73,299	96.08	2,990	3.92	460	0.62	**76,289**	**73,759**
**Chr17** **(b37)**	76,187	98.27	1,341	1.73	441	0.58	**77,528**	**76,628**
**Chr18** **(b38)**	73,375	96.83	2,404	3.17	371	0.5	**75,779**	**73,746**
**Chr18_L** **(b38)**	74,194	97.91	1,585	2.09	295	0.4	**75,779**	**74,489**
**Chr18** **(b37)**	93,004	97.97	1,923	2.03	365	0.39	**94,927**	**93,369**
**Chr19** **(b38)**	56,171	95.21	2,827	4.79	480	0.85	**58,998**	**56,651**
**Chr19_L** **(b38)**	55,897	94.74	3,101	5.26	422	0.75	**58,998**	**56,319**
**Chr19** **(b37)**	59,138	97.93	1,248	2.07	376	0.63	**60,386**	**59,514**
**Chr20** **(b38)**	64,800	96.8	2,140	3.2	399	0.61	**66,940**	**65,199**
**Chr20_L** **(b38)**	65,006	97.11	1,934	2.89	335	0.51	**66,940**	**65,341**
**Ch20** **(b37)**	64,827	97.89	1,400	2.11	275	0.42	**66,227**	**65,102**
**Chr21** **(b38)**	42,433	96.92	1,349	3.08	229	0.54	**43,782**	**42,662**
**Chr21_L** **(b38)**	42,830	97.83	952	2.17	197	0.46	**43,782**	**43,027**
**Chr21** **(b37)**	43,941	98.13	836	1.87	178	0.4	**44,777**	**44,119**
**Chr22** **(b38)**	33,336	96.77	1,114	3.23	209	0.62	**34,450**	**33,545**
**Chr22_L** **(b38)**	33,418	97	1,032	3	209	0.62	**34,450**	**33,627**
**Chr22** **(b37)**	36,132	98.16	678	1.84	207	0.57	**36,810**	**36,339**
**ChrX** (b38) *	112	96.55	4	3.45	2	1.75	**116**	**114**
AVG ** **(b38)**	**129,560**	**96.37**	**4,970**	**3.63**	**682**	**0.54**	**134,530**	**130,242**
AVG ** **(b38_** **lifted)**	**130,600**	**97.01**	**3,931**	**2.99**	**658**	**0.51**	**134,530**	**131,258**
AVG ** **(b37)**	**138,329**	**97.86**	**3,095**	**2.14**	**627**	**0.45**	**141,424**	**138,955**

* Only PAR regions ** Not considering chrX for the calculation

**Table 9.** INDEL site comparison for NA12878 between our call set and Genome in a Bottle (GIAB)-mapped to GRCh38, between the lift-over (chr*_L rows in the table) call set-mapped to GRCh38 and between the 1000 Genomes Project phase three (P3) call set and GIAB mapped to GRCh37. Results are shown for each chromosome. *‘Shared (TP)’* are the true positive variants identified in the compared call sets. *‘giab_only (FN)’* are the false negative variants identified by GIAB only. *‘Thiswork_only (FP)’* are the false positive variants identified in our call set only.

Dataset	shared (TP)	% shared (TP)	giab_only (FN)	% giab_only (FN)	Thiswork_only (FP)	%Thiswork_only (FP)	Total (GIAB)	Total thiswork_only
**Chr1** **(b38)**	24,659	63.86	13,954	36.14	3,143	11.30	38,613	27,802
**Chr1_L** **(b38)**	27,009	69.95	11,604	30.05	2,261	7.72	38,613	29,270
**Chr1** **(b37)**	25,802	73.03	9,530	26.97	2,171	7.76	35,332	27,973
**Chr2** **(b38)**	24,237	65.05	13,023	34.95	2,995	11.00	37,260	27,232
**Chr2_L** **(b38)**	26,504	71.13	10,756	28.87	2,132	7.45	37,260	28,636
**Chr2** **(b37)**	26,856	73.46	9,702	26.54	2,146	7.40	36,558	29,002
**Chr3** **(b38)**	21,768	64.95	11,745	35.05	2,530	10.41	33,513	24,298
**Chr3_L** **(b38)**	23,830	71.11	9,683	28.89	1,762	6.88	33,513	25,592
**Chr3** **(b37)**	22,495	73.94	7,930	26.06	1,693	7.00	30,425	24,188
**Chr4** **(b38)**	19,675	68.14	9,200	31.86	2,303	10.48	28,875	21,978
**Chr4_** **(b38)**	21,190	73.39	7,684	26.61	1,460	6.45	28,874	22,650
**Chr4** **(b37)**	24,275	75.40	7,921	24.60	1,694	6.52	32,196	25,969
**Chr5** **(b38)**	18,558	65.74	9,673	34.26	2,202	10.61	28,231	20,760
**Chr5_L** **(b38)**	20,330	72.01	7,901	27.99	1,436	6.60	28,231	21,766
**Chr5** **(b37)**	20,813	74.15	7,255	25.85	1,502	6.73	28,068	22,315
**Chr6** **(b38)**	20,711	65.73	10,797	34.27	2,521	10.85	31,508	23,232
**Chr6_L** **(b38)**	22,394	71.07	9,114	28.93	1,647	6.85	31,508	24,041
**Chr6** **(b37)**	20,488	74.09	7,163	25.91	1,478	6.73	27,651	21,966
**Chr7** **(b38)**	17,069	64.38	9,444	35.62	2,129	11.09	26,513	19,198
**Chr7_L** **(b38)**	18,112	68.31	8,401	31.69	1,389	7.12	26,513	19,501
**Chr7** **(b37)**	17,058	71.70	6,732	28.30	1,354	7.35	23,790	18,412
**Chr8** **(b38)**	14,387	64.00	8,093	36.00	1,761	10.91	22,480	16,148
**Chr8_L** **(b38)**	15,467	68.80	7,013	31.20	1,147	6.90	22,480	16,614
**Chr8** **(b37)**	16,164	71.64	6,400	28.36	1,207	6.95	22,564	17,371
**Chr9** **(b38)**	12,410	64.04	6,969	35.96	1,547	11.08	19,379	13,957
**Chr9_L** **(b38)**	13,476	69.54	5,903	30.46	1,149	7.86	19,379	14,625
**Chr9** **(b37)**	12,691	72.88	4,722	27.12	1,058	7.70	17,413	13,749
**Chr10** **(b38)**	15,506	64.61	8,492	35.39	1,987	11.36	23,998	17,493
**Chr10_L** **(b38)**	16,771	69.88	7,227	30.12	1,341	7.40	23,998	18,112
**Chr10** **(b37)**	15,961	73.18	5,850	26.82	1,285	7.45	21,811	17,246
**Chr11** **(b38)**	15,605	66.40	7,898	33.60	1,845	10.57	23,503	17,450
**Chr11_L** **(b38)**	17,013	72.39	6,490	27.61	1,266	6.93	23,503	18,279
**Chr11** **(b37)**	16,071	75.63	5,179	24.37	1,208	6.99	21,250	17,279
**Chr12** **(b38)**	14,366	63.28	8,335	36.72	1,854	11.43	22,701	16,220
**Chr12_L** **(b38)**	15,608	68.75	7,093	31.25	1,275	7.55	22,701	16,883
**Chr12** **(b37)**	16,042	73.25	5,859	26.75	1,334	7.68	21,901	17,376
**Chr13** **(b38)**	12,631	68.28	5,869	31.72	1,485	10.52	18,500	14,116
**Chr13_L** **(b38)**	13,634	73.70	4,866	26.30	1,039	7.08	18,500	14,673
**Chr13** **(b37)**	12,990	76.03	4,096	23.97	970	6.95	17,086	13,960
**Chr14** **(b38)**	10,344	64.71	5,640	35.29	1,338	11.45	15,984	11,682
**Chr14_L** **(b38)**	11,268	70.50	4,716	29.50	1,024	8.33	15,984	12,292
**Chr14** **(b37)**	10,764	74.57	3,670	25.43	896	7.68	14,434	11,660
**Chr15** **(b38)**	8,770	64.28	4,874	35.72	1,052	10.71	13,644	9,822
**Chr15_L** **(b38)**	9,746	71.43	3,898	28.57	792	7.52	13,644	10,538
**Chr15** **(b37)**	9,265	74.66	3,145	25.34	728	7.29	12,410	9,993
**Chr16** **(b38)**	4,662	61.17	2,959	38.83	704	13.12	7,621	5,366
**Chr16_L** **(b38)**	5,233	68.67	2,388	31.33	520	9.04	7,621	5,753
**Chr16** **(b37)**	8,409	70.44	3,529	29.56	837	9.05	11,938	9,246
**Chr17** **(b38)**	8,053	60.29	5,303	39.71	1,136	12.36	13,356	9,189
**Chr17_L** **(b38)**	8,977	67.21	4,379	32.79	867	8.81	13,356	9,844
**Chr17** **(b37)**	8,866	70.94	3,632	29.06	828	8.54	12,498	9,694
**Chr18** **(b38)**	7,618	67.20	3,718	32.80	928	10.86	11,336	8,546
**Chr18_L** **(b38)**	7,915	69.82	3,421	30.18	581	6.84	11,336	8,496
**Chr18** **(b37)**	9,482	72.12	3,666	27.88	689	6.77	13,148	10,171
**Chr19** **(b38)**	6,090	56.81	4,630	43.19	896	12.83	10,720	6,986
**Chr19_L** **(b38)**	6,620	61.75	4,100	38.25	694	9.49	10,720	7,314
**Chr19** **(b37)**	6,638	66.14	3,398	33.86	701	9.55	10,036	7,339
**Chr20** **(b38)**	6,430	62.55	3,849	37.45	823	11.35	10,279	7,253
**Chr20_L** **(b38)**	6,744	65.61	3,535	34.39	559	7.65	10,279	7,303
**Ch20** **(b37)**	6,435	68.82	2,915	31.18	528	7.58	9,350	6,963
**Chr21** **(b38)**	4,752	67.60	2,278	32.40	547	10.32	7,030	5,299
**Chr21_L** **(b38)**	5,144	73.17	1,886	26.83	350	6.37	7,030	5,494
**Chr21** **(b37)**	5,104	76.49	1,569	23.51	330	6.07	6,673	5,434
**Chr22** **(b38)**	3,399	60.02	2,264	39.98	479	12.35	5,663	3,878
**Chr22_L** **(b38)**	3,764	66.48	1,898	33.52	353	8.57	5,662	4,117
**Chr22** **(b37)**	4,072	69.91	1,753	30.09	362	8.16	5,825	4,434
**ChrX** (b38)*	15	53.57	13	46.43	5	25.00	28	20
AVG** **(b38)**	**13,259**	**64.23**	**7,228**	**35.77**	**1,646**	**11.23**	**20,487**	**14,905**
AVG** **(b38** **_lifted)**	**14,398**	**69.76**	**6,089**	**30.24**	**1,138**	**7.52**	**20,487**	**15,536**
AVG** **(b37)**	**14,397**	**72.84**	**5,255**	**27.16**	**1,136**	**7.45**	**19,653**	**15,534**

* Only PAR regions ** Not considering chrX for the calculation

In order to characterize the profile of the GIAB NA12878 SNV sites that were missing in our call set but were present in the lift-over call set, we examined if we had evidence in any of our intermediate files of the presence of these sites, and if so, we looked for an explanation for these sites being discarded. The result of this analysis is shown in Table 10, where we can see that most of the sites that were missing (68.7% on average across all the autosomes) were discarded because of the filtering sensitivity cutoff used with VQSR ApplyRecalibrator ( --ts_filter_level 99.5) during the final filtering step of the consensus call set.

In the case of INDELs, we identified on average 64.2% of the INDEL sites that are also present in GIAB. This percentage is lower than the 73% obtained for the comparison between P3 and GIAB and lower than the 69.8% for the comparison of the lift-over with GIAB. This is possibly due to the fact that P3 used a higher number of algorithms specialized in the identification of INDELs than the ones used in this work

**Table 10.** Analysis of the number of GIAB NA12878 SNV sites present in the lift-over call set not identified in our call set. ‘False negatives’ column contains the count of GIAB NA12878 SNV sites that were identified in the lift-over call set and not in our work. ‘VQSRTrancheSNP99.50to99.9’ column contains the count of false negative sites that were filtered out in our work assigned to the 99.5-99.9 quality tranche by VQSR. ‘VQSRTrancheSNP99.90to100.0’ column contains the count of false negative sites that were filtered out in our work assigned to the 99.9-100.0 quality tranche by VQSR. The higher the tranche, the higher the sensitivity and the lower the specificity of our call set. ‘% explained’ column contains the percentage of false negatives that were discarded in our work by the VQSR filtering procedure.

Dataset	False negatives	VQSRTrancheSNP99.50to99.90	VQSRTrancheSNP99.90to100.00	% explained
**Chr1**	5,224	3,666	138	72.82
**Chr2**	5,071	3,476	75	70.03
**Chr3**	4,635	3,198	92	70.98
**Chr4**	4,592	3,274	98	73.43
**Chr5**	3,949	2,808	66	72.78
**Chr6**	4,916	3,489	123	73.47
**Chr7**	3,462	2,338	78	69.79
**Chr8**	3,246	2,155	65	68.39
**Chr9**	2,652	1,750	42	67.57
**Chr10**	2,893	1,870	64	66.85
**Chr11**	3,896	2,768	91	73.38
**Chr12**	2,802	1,937	69	71.59
**Chr13**	2,024	1,261	32	63.88
**Chr14**	2,177	1,463	48	69.41
**Chr15**	1,643	1,028	33	64.58
**Chr16**	977	528	17	55.78
**Chr17**	1,359	835	37	64.16
**Chr18**	1,261	821	25	67.09
**Chr19**	1,401	929	38	69.02
**Chr20**	1,051	623	18	60.99
**Chr21**	728	477	10	66.9
**Chr22**	628	477	10	77.55
**AVG %**	2,753.95	1,871.41	57.68	68.66

### Comparison of updated clinical loci

**Figure 4.**
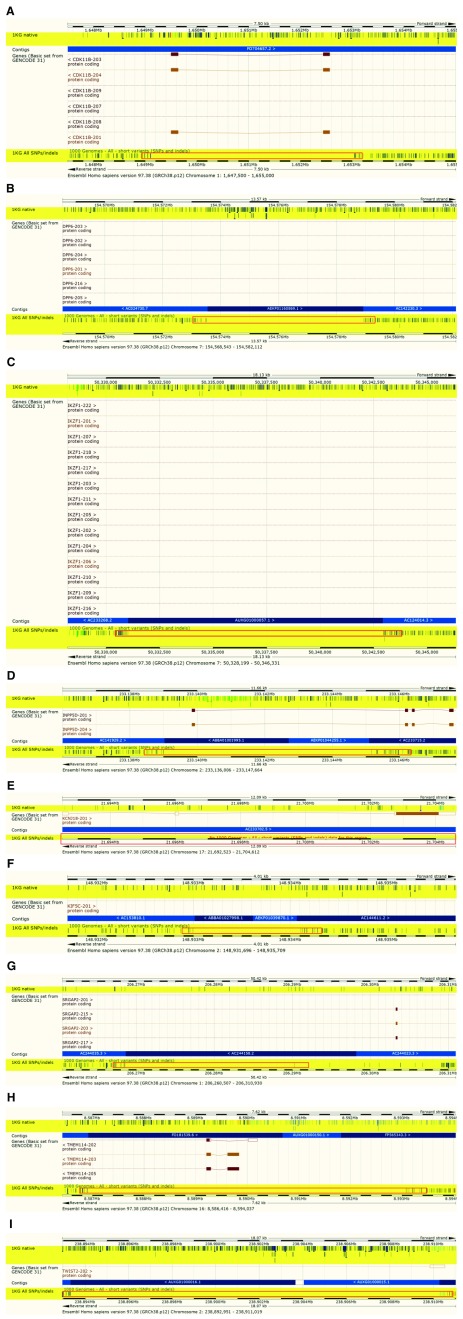
Variants in regions containing clinically relevant genes that had coding sequence splits over assembly gaps in GRCh37 that have been filled in GRCh38. ‘ *1KG native*’: call set presented in this work; ‘ *1KG All SNPs/indels*’: lift-over call set.

We further compared our call set and the lift-over call set in the regions identified by Schneider *et al*. ^3^ with assembly updates in GRCh38. The authors looked at the intersection of the transcripts having problems in the alignment with GRCh37 with two lists of clinically relevant genes: a set of genes enriched for *de novo* loss of function mutations identified in Autism Spectrum Disorder (n = 1003) ^22^ and a collection of genes preliminarily proposed for the development of a medical exome kit (n = 4623) ( https://www.genomeweb.com/diagnostics/emory-chop-harvard-develop-medical-exome-complete-coverage-5k-disease-associated-genes). Schneider *et al*. ^3^ show in their analysis that there were 14 genes from these two lists for which the alignment issues disappear when GRCh38 is used (see Table 11). Unsurprisingly, when viewing these regions, we see an absence of variation in the lift-over while calls have been made in the *de novo* analysis. This is illustrated in Figure 4.

**Table 11.** Autism Spectrum Disorder genes 22 and Medical Exome Kit Genes ( https://www.genomeweb.com/sequencing/emory-chop-harvard-develop-medical-exomekit-complete-coverage-5k-disease-associ) that had transcript alignment issues with GRCh37 but not with GRCh38. RefSeq release version 71. ‘ *True*’ indicates presence in the relevant gene list. ‘ *False*’ indicates absence.

GeneID	GeneSymbol	MedicalExome	[ 22]
984	CDK11B	False	True
10320	IKZF1	True	True
3635	INPP5D	True	True
3800	KIF5C	False	True
102724631	POTEB3	False	True
23380	SRGAP2	False	True
1804	DPP6	True	False
100134444	KCNJ18	True	False
5645	PRSS2	True	False
374462	PTPRQ	True	False
259291	TAS2R45	True	False
283953	TMEM114	True	False
117581	TWIST2	True	False

### Novel GRCh38 contigs

We have also analysed the number of SNV variants located in the new contigs added to GRCh38 to update sequence or fill gaps present in GRCh37. The coordinates for these new contigs were obtained using UCSC’s table browser ^23^, retrieving the data for the Hg19Diff track from the hg38ContigDiff primary table. Only the records having a score=0, which correspond to the coordinates of the new contigs added to GRCh38 to update sequence or fill gaps present in GRCh37 were considered. Table 12 shows the comparison of the number of SNVs identified in the new contigs with the number in regions that were already present in GRCh37. We can see in these tables that the percentage of SNVs in the new GRCh38 contigs is higher in our call set (55.7% vs 44.3%) than in the lift-over call set, whereas the percentage is lower (48% vs 52%) for the rest of the genome.

**Table 12.** Number of biallelic SNVs in our call set (‘ *This_work*’) and in the ‘ *Lift-over*’ call set. ‘ *novel*’ represent the new contigs added to GRCh38 whereas ‘ *existing*’ represent the rest of the genomic regions that were already present in GRCh37.

Region	This_work	Lift-over	Total
**novel**	1,019,976 (55.7%)	811,817 (44.3%)	1,831,793 (100%)
**existing**	70,809,835 (48%)	76,588,820 (52%)	147,398,655 (100%)

**Figure 5.**
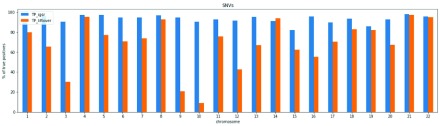
Percentage of SNVs that are true positives in the comparison with the NA12878 call set from GIAB for contigs added to GRCh38 across the different autosomes. ‘ *TP_igsr*’ is the percentage of true positives for our call set. ‘ *TP_liftover*’ is the percentage of true positives for the lift-over call set.

***Concordance with Genome in a bottle (GIAB) NA12878 in novel regions***. We have also examined the overlap for biallelic SNV sites identified in sample NA12878 between the GIAB sites on the new GRCh38 contigs, our call set and the lift-over call set. Figure 5 has a barplot with the percentage of sites overlapping with GIAB and we can see that this percentage is greater in our call set in all the autosomes except chromosome 14, reaching percentages of 90% in our call set and only 9% in the lift-over call set for chromosome 10. This demonstrates that calling directly on GRCh38 can produce calls that are more reliable than a lift-over for novel regions.

### Call set performance summary

The benchmarking results show that, unsurprisingly, given the breadth of callers and extensive integration and filtering work, that phase three of 1000 Genomes Project performed best in comparison to GIAB on GRCh37. Further, we see only slightly diminished performance from the lift-over, when judging on genome wide metrics. Given that only some regions of the primary assembly have altered and that the benchmark (GIAB), like the original data set, does not interact with the alts, this may also not be wholly surprising. This picture, however, does change when looking in detail at improved regions of the assembly. Here, as expected, we see regions where the liftover contains no calls, because the sequence was not in GRCh37 and, therefore, could not possibly be called on - although our work demonstrates that calls are made.

In assessing the *de novo* call set, it seems that the reduced range of callers and simplified methodology, combined with a conservative filtering approach, mean that, relative to phase three, the GRCh38 *de novo* call set has slightly reduced sensitivity. However, its performance is of a similar order to those of the original phase three call set and the lift-over, while providing a consistent analysis of the data across the improved assembly, including some clinically significant novel regions where calls were not previously made.

As sequencing has progressed since the 1000 Genomes Project, it is also interesting to compare to modern data types. We looked at the calls recently released by the New York Genome Center (NYGC) for NA12878 which are part of a GATK HaplotypeCaller call set for the 2504 member phase three panel, which has been resequenced to 30x coverage ( http://ftp.1000genomes.ebi.ac.uk/vol1/ftp/data_collections/1000G_2504_high_coverage/). We retrieved the NA12878 calls and compared them to the GIAB GRCh38 call set. The average percentage of SNV sites identified by GATK HC in the high coverage data across all the chromosomes that were also present in GIAB represents 94.2% of the total GIAB sites, which is slightly lower than the 96.4% obtained in our work. In the case of INDEL sites, GATK HC identified an average of 42.1% of the total GIAB INDEL sites, which is lower than the 64.2% that we obtained for our call set. We anticipate that, as analysis of the high coverage data progresses, those outputs will replace the work described here but note that our approach achieves comparable results to those of a modern production pipeline.

## Data availability

The variants resulting from this work are available in the European Variation Archive. Accession number PRJEB31735.

This call set is also available from the International Genome Sample Resource (IGSR) ^4^ at: http://ftp.1000genomes.ebi.ac.uk/vol1/ftp/data_collections/1000_genomes_project/release/20190312_biallelic_SNV_and_INDEL/.

## Software availability

**Table:** 

Task	Codebase	Documentation	Licence	DOI	Ref.
**eHive** **(workflow** **system)**	https://github.com/ Ensembl/ensembl-hive	https://ensembl-hive. readthedocs.io/en/ version-2.5/	Apache 2.0	NA	17
**BAM quality** **control**	https://github.com/igsr/igsr_ analysis/tree/v1.0.0/PyHive/ BamQC	**WGS BAM QC: ** https:// igsr-analysis.readthedocs. io/en/latest/workflows/ wgs_bamqc_pipeline.html **WES BAM QC:** https:// igsr-analysis.readthedocs. io/en/latest/workflows/ wes_bamqc_pipeline.html	Apache 2.0	http://doi.org/10.5281/ zenodo.2573911	18
**Variant** **discovery**	https://github.com/EMBL- EBI-GCA/reseqtrack/ tree/master/modules/ ReseqTrack/Hive	https://github.com/EMBL- EBI-GCA/reseqtrack/ blob/master/docs/ variantcalling_pipeline.txt	Apache 2.0	https://doi.org/10.5281/ zenodo.2573969	19
**Variant** **filtering**	https://github.com/igsr/igsr_ analysis/tree/v1.0.0/PyHive/ PipeConfig/FILTER	**BCFtools WGS variant** **filtering pipeline: ** https:// igsr-analysis.readthedocs. io/en/latest/workflows/ bcftools_wgs_filtering_ pipeline.html **BCFtools WES variant** **filtering pipeline:** https:// igsr-analysis.readthedocs. io/en/latest/workflows/ bcftools_wes_filtering_ pipeline.html **Freebayes variant** **filtering pipeline:** https:// igsr-analysis.readthedocs. io/en/latest/workflows/ freebayes_filtering_ pipeline.html **GATK variant filtering** **pipeline:** https://igsr- analysis.readthedocs. io/en/latest/workflows/ gatk_vc_filtering_pipeline. html	Apache 2.0	http://doi.org/10.5281/ zenodo.2573911	18
**Variant** **integration**	https://github.com/igsr/igsr_ analysis/blob/v1.0.0/PyHive/ PipeConfig/INTEGRATION/ VCFIntegrationGATKUG.pm	https://igsr-analysis. readthedocs.io/en/latest/ workflows/consensus_ callset_pipeline.html	Apache 2.0	http://doi.org/10.5281/ zenodo.2573911	18
**Phasing**	https://github.com/igsr/igsr_ analysis/blob/v1.0.0/PyHive/ PipeConfig/INTEGRATION/ PHASING.pm	https://igsr-analysis. readthedocs.io/en/latest/ workflows/phasing_ pipeline.html	Apache 2.0	http://doi.org/10.5281/ zenodo.2573911	18
**Benchmarking** **using Genome** **in a Bottle**	https://github.com/igsr/igsr_ analysis/blob/v1.0.0/scripts/ VCF/QC/compare_with_giab. nf	https://igsr-analysis. readthedocs.io/en/latest/ workflows/compare_with_ giab_pipeline.html	Apache 2.0	http://doi.org/10.5281/ zenodo.2573911	18

## References

[1] 1000 Genomes Project Consortium, Auton A Brooks LD: A global reference for human genetic variation. *Nature.* 2015;526(7571):68–74. 10.1038/nature15393 26432245 PMC4750478

[2] Zheng-Bradley X Flicek P: Applications of the 1000 Genomes Project resources. *Brief Funct Genomics.* 2017;16(3):163–170. 10.1093/bfgp/elw027 27436001 PMC5439288

[3] Schneider VA Graves-Lindsay T Howe K: Evaluation of GRCh38 and *de novo* haploid genome assemblies demonstrates the enduring quality of the reference assembly. *Genome Res.* 2017;27(5):849–864. 10.1101/gr.213611.116 28396521 PMC5411779

[4] Fairley S Lowy-Gallego E Perry E: The International Genome Sample Resource (IGSR) collection of open human genomic variation resources. *Nucleic Acids Res.* 2019; [cited 7 Oct 2019]. 10.1093/nar/gkz836 31584097 PMC6943028

[5] Cunningham F Achuthan P Akanni W: Ensembl 2019. *Nucleic Acids Res.* 2019;47(D1):D745–D751. 10.1093/nar/gky1113 30407521 PMC6323964

[6] Zheng-Bradley X Streeter I Fairley S: Alignment of 1000 Genomes Project reads to reference assembly GRCh38. *Gigascience.* 2017;6(7):1–8. 10.1093/gigascience/gix038 28531267 PMC5522380

[7] 1000 Genomes Project Consortium, Abecasis GR Altshuler D: A map of human genome variation from population-scale sequencing. *Nature.* 2010;467(7319):1061–1073. 10.1038/nature09534 20981092 PMC3042601

[8] 1000 Genomes Project Consortium, Abecasis GR Auton A: An integrated map of genetic variation from 1,092 human genomes. *Nature.* 2012;491(7422):56–65. 10.1038/nature11632 23128226 PMC3498066

[9] Maccari G Robinson J Ballingall K: IPD-MHC 2.0: an improved inter-species database for the study of the major histocompatibility complex. *Nucleic Acids Res.* 2017;45(D1):D860–D864. 10.1093/nar/gkw1050 27899604 PMC5210539

[10] Jun G Flickinger M Hetrick KN: Detecting and estimating contamination of human DNA samples in sequencing and array-based genotype data. *Am J Hum Genet.* 2012;91(5):839–848. 10.1016/j.ajhg.2012.09.004 23103226 PMC3487130

[11] McKenna A Hanna M Banks E: The Genome Analysis Toolkit: a MapReduce framework for analyzing next-generation DNA sequencing data. *Genome Res.* 2010;20(9):1297–1303. 10.1101/gr.107524.110 20644199 PMC2928508

[12] Zook JM Chapman B Wang J: Integrating human sequence data sets provides a resource of benchmark SNP and indel genotype calls. *Nat Biotechnol.* 2014;32(3):246–251. 10.1038/nbt.2835 24531798

[13] Tan A Abecasis GR Kang HM: Unified representation of genetic variants. *Bioinformatics.* 2015;31(13):2202–2204. 10.1093/bioinformatics/btv112 25701572 PMC4481842

[14] Browning SR Browning BL: Rapid and accurate haplotype phasing and missing-data inference for whole-genome association studies by use of localized haplotype clustering. *Am J Hum Genet.* 2007;81(5):1084–1097. 10.1086/521987 17924348 PMC2265661

[15] Delaneau O Marchini J Zagury JF: A linear complexity phasing method for thousands of genomes. *Nat Methods.* 2011;9(2):179–181. 10.1038/nmeth.1785 22138821

[16] Delaneau O Marchini J 1000 Genomes Project Consortium, : Integrating sequence and array data to create an improved 1000 Genomes Project haplotype reference panel. *Nat Commun.* 2014;5: 3934. 10.1038/ncomms4934 25653097 PMC4338501

[17] Severin J Beal K Vilella AJ: eHive: an artificial intelligence workflow system for genomic analysis. *BMC Bioinformatics.* 2010;11:240. 10.1186/1471-2105-11-240 20459813 PMC2885371

[18] Lowy E GabeAldam Fairley S: igsr/igsr_analysis: First release of code (Version v1.0.0). *Zenodo.* 2019 10.5281/zenodo.2573911

[19] istreeter Richardson D HollyZB: EMBL-EBI-GCA/reseqtrack: zenodo (Version zenodo). *Zenodo.* 2019 10.5281/zenodo.2573969

[20] Patterson M Marschall T Pisanti N: WhatsHap: Weighted Haplotype Assembly for Future-Generation Sequencing Reads. *J Comput Biol.* 2015;22(6):498–509. 10.1089/cmb.2014.0157 25658651

[21] Di Tommaso P Chatzou M Floden EW: Nextflow enables reproducible computational workflows. *Nat Biotechnol.* 2017;35(4):316–319. 10.1038/nbt.3820 28398311

[22] Samocha KE Robinson EB Sanders SJ: A framework for the interpretation of *de novo* mutation in human disease. *Nat Genet.* 2014;46(9):944–950. 10.1038/ng.3050 25086666 PMC4222185

[23] Karolchik D Hinrichs AS Furey TS: The UCSC Table Browser data retrieval tool. *Nucleic Acids Res.* 2004;32(Database issue):D493–6. 10.1093/nar/gkh103 14681465 PMC308837

[24] Poznik GD Xue Y Mendez FL: Punctuated bursts in human male demography inferred from 1,244 worldwide Y-chromosome sequences. *Nat Genet.* 2016;48(6): 593–599. 10.1038/ng.3559 27111036 PMC4884158

