# Supplementary material for: Variant calling on the GRCh38 assembly with the data from phase three of the 1000 Genomes Project
Source: Wellcome Open Res. 2019 Dec 30;4:50. Originally published 2019 Mar 11. [Version 2] doi: 10.12688/wellcomeopenres.15126.2 (PMC7059836; doi:10.12688/wellcomeopenres.15126.2)
Supplement: Peer review history

# Peer review: Reviewer response for version 2

**DOI:** 10.21956/wellcomeopenres.17129.r37471

**Published:** 2020-03-04

**Authors:** Deanna M. Church

**Affiliations:** 1 Inscripta, Inc., Boulder, CO, USA

The authors have revised this manuscript nicely. While I still think the lack of calls on the X and Y is unfortunate, the authors do a good job of explaining this. The addition of indels and phasing is welcome. The manuscript is much clearer and easier to follow, and makes a better case for why going to the trouble of reanalyzing data rather than performing a lift-over is worth the effort.

I confirm that I have read this submission and believe that I have an appropriate level of expertise to confirm that it is of an acceptable scientific standard.

# Peer review: Reviewer response for version 2

**DOI:** 10.21956/wellcomeopenres.17129.r37455

**Published:** 2020-01-16

**Authors:** Augusto Rendon

**Affiliations:** 1 Genomics England, London, UK; 2 University of Cambridge, Cambridge, UK

The authors have revised the document and added the INDEL call set. 

 In my opinion, the authors have provided sufficient data and analyses to enable the reader to understand the caveats associated with the data prepared by them. 

 I have one small remaining concern. I would like it if the authors clarify the behaviour of their processing when dealing with multinucleotide variants (MNVs). Are they excluded? If not, then the normalisation and merging approaches may render spurious results.

I confirm that I have read this submission and believe that I have an appropriate level of expertise to confirm that it is of an acceptable scientific standard.

# Peer review: Reviewer response for version 1

**DOI:** 10.21956/wellcomeopenres.16504.r35054

**Published:** 2019-04-10

**Authors:** Augusto Rendon

**Affiliations:** 1 Genomics England, London, UK; 2 University of Cambridge, Cambridge, UK

**Summary**

 The authors present a new call set from the 1000 genomes project. This time the call set is a fresh recall of the data against GRCh38. The call set is only composed of biallelic SNPs (biallelic in this study). Previous variant call sets on GRCh38 had been lifted over from their GRCh37 native counterparts. The study identifies SNPs by first calling variants with several algorithms, creating an union set and then explicitly genotyping these sites across the complete data set. The genotypes are then phased. A comparison with GIAB data for NA12878 is performed to assess the sensitivity and specificity of the call set.

**General observations**

 A native call set of the 1000 genomes project data on GRCh38 is quite an important effort. These data have many important downstream uses including clinical genomics uses for variant filtering and population genomics studies. Notwithstanding the importance of this data set, I have issues with the data note as it stands. I think the paper simply described what the authors did to generate these data but makes little effort to explain why it was done in this way. The latter is important to gain confidence in the call set.

 As a user of the data I would look to be satisfied with the quality of the call set. This is particularly important as several other large scale projects have released allele frequencies of many thousands of deep sequenced whole genomes (e.g. Topmed and Gnomad). In parallel, frameworks for assessing the analytical performance of variant calls have been proposed by GIAB and GA4GH and recently published. Truth sets for several important samples beyond the NA12878 have been released; including the ability to assess to how well phased the data sets was. As explained in the specific comments below, I believe more work should have been done to convince the reader that sufficient due diligence has been committed to this data set.

 From the perspective of having a native call set on GRCh38 it is a shame that there is very little to show the potential benefits of this call set on GRCh38. There is no mention [or I really missed it] about how alternative haplotypes were handled and the implications of having variants in alternative haplotypes. The data release also ignores any non diploid areas of the genome.

 From a methodological perspective, the tool chain feels outdated with BCFtools and GATK being at least 2 years old. Thresholds are widely used but little work is done to explain how these thresholds are determined. I assume that the parameters used in the tools themselves, these are standard or perhaps defined in previous iterations of the project. However, the filtering thresholds in Tables 1-4, at least from the reading, sounds like they were plucked out of thin air.

 On a more positive note, I would like to commend authors for the great effort placed to make available the code, organising it, document it, and making this data set reproducible.

**Specific observations**

*Tool chain is really outdated*. Is this because there have been little changes for the specific algorithms used (mpileup and unified genotyper?). I assume that many improvements and bugfixes have appeared in the last two years plus since these versions were released.
*Demonstrating improvements on GRCh38 and why it is better than the lift over*. I missed some figure showing how this was an improvement. For example a comparison of the lifted over and the native call set. Are there regions of the genome where they perform different? Was it worth the effort? What about the ALTs, have we now better allele frequencies on these regions? How do they affect the frequencies on the corresponding frequencies on the primary assembly?
*Variant normalisation*. This is glossed over in the text and little is said about how it was performed. In my experience this step often introduces difficult tradeoffs. Please expand on this.
*Benchmarking*. This area is quite lacking here. I understand that you are only looking at biallelic sites so comparison with a truth set is easier. However, standards to doing this have existed for over a year and recently published. They are based on comparing at the haplotype level and not the site level.
*Phasing*. These standards also enable comparing the phasing accuracy. Given the effort placed here to phase the genotypes, it would be helpful to also benchmark the phasing data.
*Union set*. I would have loved to see a figure that shows how the various variant callers contributed to the union call set. Is there one that is unnecessary? Is there one that is responsible for many of the false positives?
*Analytical performance*. "Taken together, these results demonsrate both the high sensitivity and high specificity of our callset". You seem to have less TP and more FN than in the 37 release. Now a days these numbers do not represent high sensitivity and specificity (at least with 30X genomes). At least a more nuanced discussion is required here. This is strongly linked to the thresholds you chose for various filtering steps.
*Truth sets*. It would be very helpful to add further truth sets, for example for the Ashkenazim and Chinese trios.
*Joint calling*. The approach here was to do single sample calling, assembling a union set and then genotyping those sites. Could the authors please explain why this approach as opposed to joint calling. I would assume that for low coverage genomes, joint calling may be more powerful as it can leverage information across more samples.

I confirm that I have read this submission and believe that I have an appropriate level of expertise to state that I do not consider it to be of an acceptable scientific standard, for reasons outlined above.

## References

[1] : Best practices for benchmarking germline small-variant calls in human genomes. *Nat Biotechnol*.2019; 10.1038/s41587-019-0054-x 10.1038/s41587-019-0054-x 30858580PMC6699627

[2] : An open resource for accurately benchmarking small variant and reference calls. *Nat Biotechnol*.2019; 10.1038/s41587-019-0074-6 10.1038/s41587-019-0074-6 30936564PMC6500473

# Response

**Published:** 2019-12-06

**Authors:** Ernesto Lowy

First, we would like to thank the reviewer for the feedback that has been provided and for giving this work their attention.

In the general observations the reviewer notes that the data note is limited to a description of what was done, without significant discussion of the rationale behind the approach. We note that this is a data note, intended to describe a data set and how it was produced, however, we have also amended the text to include more detail relating to the rationale behind our approach.

Also, in general observations, the reviewer highlights issues relating to data quality: other call sets, the limitation of comparing to only NA12878 and benchmarking phasing. While other call sets, such as TOPmed and gNOMAD exist, the 1000 Genomes Project data set remains unique in terms of its composition of populations and that all data can be accessed to the base pair level. With regard to other truth sets, beyond NA12878, we were unable to locate “gold-standard” data for any other samples in our data set. With regard to phasing, we have used the WhatsHap program to assess this and added the results to the manuscript.

With regard to GRCh38, our aim was not to demonstrate the superiority of GRCh38 but rather to provide a resource for those wishing to use that assembly. We believe the benefits of the assembly have been demonstrated by Schneider *et al*.

We have amended the text to make it clearer that calling was not done on alternate loci. The text has also been amended to describe the intention of using the data note format to release and describe data early, with the intention being to revisit elements not included in this set.

With regard to the tool chain, this reflects the volume of compute involved in this work. It was around two years ago that this work commenced. The final stage of the pipeline alone takes around six months to run, even with access to generous compute resources.

The text has been amended to include information on threshold selection.

With regard to the detailed points:

**1) Tool chain is really outdated**

Software versions reflect the length of time required to run this compute, as noted above. 

**2) Demonstrating improvements on GRCh38 and why it is better than the lift over**

As noted above, our intention was not to demonstrate that GRCh38 was the better assembly. We consider that this has been done previously by Schneider *et al*. We have added a comparison with the liftover.

**3) Variant normalisation**

We have updated the text to include further information on this.

**4) Benchmarking**

We note that hap.py was published after this work was submitted. We were unable, however, to establish from the manuscript how it could be used to improve upon the existing benchmarking. The summary provided in Figure 1 (https://www.nature.com/articles/s41587-019-0054-x) indicates that it wraps tools for arriving at consistent representation of variants (handled in the normalisation steps of our pipeline at the point of producing the consensus call set) and then produces a “standardised” report, providing similar metrics to those we present. From this, it appears to provide similar functionality to steps already present in our work. Our attempt to contact the authors for more information on this was not successful.

With regard to our decision to use a “truth set”, our belief is that comparing to an independently produced “gold-standard” is a valuable benchmarking strategy.

We have extended the benchmarking using WhatsHap.

**5) Phasing**

This has been done with WhatsHap and the results added. As noted above, our attempt to contact the author of hap.py to establish how it could be used to benchmark phasing was sadly unsuccessful.

**6) Union set**

We have added the requested figure.

**7) Analytical performance**

These statements were made in the context of comparison with the phase three call set. The text has been amended. We have also compared with an initial analysis of new 30x coverage data produced by standard pipelines at NYGC. Based on our benchmark, the performance of our call set is slightly better. We agree that filtering has an impact here.

**8) Truth sets**

Our calling approach used low-coverage and exome data from the period of approximately 2008 to 2012 to perform joint genotyping. We believe that there would be questions about the validity of trying to benchmarking our results with samples that are simultaneously (1) not part of one of our populations, (2) have different relatedness to other samples in the population, and (3) have different data types available for variant calling. This excludes the Ashkenazi samples as a suitable benchmark. For the Han Chinese samples, we could not locate data that matches the profile of that for our samples. We have updated the text in an effort to improve the discussion of issues relating to benchmarking.

**9) Joint calling**

This did use joint calling. The text has been amended to make this clearer.

# Peer review: Reviewer response for version 1

**DOI:** 10.21956/wellcomeopenres.16504.r35051

**Published:** 2019-04-08

**Authors:** Deanna M. Church

**Affiliations:** 1 Inscripta, Inc., Boulder, CO, USA

**Summary**

 In the work entitled 'Variant calling on the GRCh38 assembly with the data from phase three of the 1000 Genomes Project', Lowy-Gallego *et al.* describe their effort to re-analyze the 1000 genomes data on the current GRCh38 assembly. They do not perform a full variant analysis, but rather release a set of biallelic SNVs as a preliminary variant call set. They compare this variant set to the Genome in a Bottle (GIAB) variant calls on the sample NA12878.

**High Level Comments**

 It is great to see efforts to update important datasets onto the current human reference assembly, GRCh38. As the authors note, the GRCh38 reference represents a substantial improvement over the GRCh37 reference, but the lack of GRCh38 based annotation has hindered adoption of this version of the reference assembly. The authors go on to discuss why 'lift-over' based approaches are inadequate, which motivated this work. I agree that 'lift-over' based approaches are inadequate, but I find the results presented in this manuscript unconvincing with respect to this assertion.

 The authors spend significant space in the introduction on both explaining the improvements in GRCh38, including the addition of alternate loci, but then put no effort into demonstrating why these are valuable. Additionally, the authors spend time discussing why 'lift-over' based approaches are inadequate, but then do no comparisons to show why their *de novo* approach is an improvement.

 While I believe this work is important, I feel the authors fail to make the point of why doing this *de novo* analysis on the GRCh38 reference is important.

**Detailed Comments**

 1. **Explanation of why 'lift-over' approaches have limitations: **I agree with the statement that 'lift-over' is inadequate. However, the description of this on page 1 is not clear. Statement 1 'they rely on an equivalent region existing in the new genome, so new sequence in the improved assembly is effectively excluded' confounds two points. Regions that are present on the old assembly but no the new one will be excluded from a 'lift-over' approach. Additionally, sequence that is new on the updated reference will also be omitted - but these are two separate cases.

 Point 2, relating two alignments also confounds multiple issues. Yes - correct alignments are key to the 'lift-over' approach, but there are two 'bad alignment' cases. The case I **think** the manuscript is referring to is a case where increased diversity in one version of the assembly can confound alignments (that is, sequence change). The other relevant case is the addition of paralogous sequence to one assembly that is missing from the other. This can lead to a locus aligning to a paralogous region rather then the equivalent locus (I have seen examples of this), that can also lead to incorrect 'lift-over'. Point three in this statement is a clear statement, but the authors provide no evidence to actually support this.

 2. **The authors only provide biallelic SNPs:** I can see the utility in concentrating on a restricted set of variants, but only if this set of data are actually used to demonstrate the value of *de novo* analysis over 'lift-over', which was not done in this manuscript. On page 3, the authors state that "These represent the major part of the SNVs present in the human genome." but I'd like more hard numbers on this. What percentage of all SNVs do the biallelics represent? What percentage of all variation do they represent?

 3. **Page 3, Quality control of alignment files:** Are the steps presented here just the differences from the original protocol? I think this is OK, but it is not clear from reading the manuscript if this is the full set of steps or just the differences.

 4. **Variant discovery:** Why did you use the variant calling tools you chose?

 5. **Variant Filtering:** The omission of variants from the sex chromosomes seems like a significant omission and limits the use of this dataset.

 6. **Data set validation:** I have significant concerns here. I understand why NA12878 was used for some validation. However, my understanding is that the GIAB dataset does not take the alternate loci into account in their variant calling, while this manuscript tries to take advantage of these sequences - how did this impact the comparison? For example, I would predict more conflicts in regions where alt-loci exist in GRCh38. Does this occur?

 I am also not convinced that accuracy on NA12878 really translates well to other samples, particularly non-European samples (as NA12878 has a European ancestry). Will the accuracy really extend to non-European samples? Also, my reading of Table 5 is that this dataset performs slightly worse than the GRCh37 call set. This does not do a lot to convince this reader that the work of doing the re-analysis is worthwhile - and I'm a believer, based on previous work I have been a part of! I have some concerns that this may be due to improvements in the new call set (due to the inclusion of the alts and more complex decoy) but it takes some significant work to track this down. There are examples of this kind of analysis ^1^. The authors should also clearly state what fraction of the genome they are able to assess using this method.

 7. **Omitted analysis:** The authors discuss the value of the improved reference in the introduction, but then do nothing to show the value of the alternate loci. How many new variants are identified on these loci? How does the inclusion of these sequences change variant calling on the primary?

 Perhaps most disappointing is that there is no analysis of how the *de novo* is an improvement over 'lift-over' approaches. How do the *de novo* variant calls compare to the 'lift-over' calls? Without this analysis, it is unclear to me that anyone would be convinced that doing the *de novo* call approach is worth the effort.

 Lastly, the authors miss the opportunity to do an accuracy comparison by looking at the regions of the reference comprised of the 'ABC' clones. These are fosmid libraries constructed from several of the samples that went into the 1000 genomes project. These provide a great test bed for both looking at variant calls (any call in this region should be heterozygous or hemizygous as the reference sequence represents one valid haplotype in the sample being analyzed) and also allows for the confirmation of the local haplotype assertions.

I confirm that I have read this submission and believe that I have an appropriate level of expertise to state that I do not consider it to be of an acceptable scientific standard, for reasons outlined above.

## References

[1] : Resolving the full spectrum of human genome variation using Linked-Reads. *Genome Res*.29(4) : 10.1101/gr.234443.118 635-645 10.1101/gr.234443.118 30894395PMC6442396

# Response

**Published:** 2019-12-06

**Authors:** Ernesto Lowy

First, we would like to thank the reviewer for the feedback that has been provided and for giving this work their attention.

We note the high level comments are broken down into detailed points and addressed with detailed comments below. We have further updated the manuscript in an effort to improve clarity where it was indicated this was lacking. We also provided the requested information comparing the generated call set with the lift-over and included assorted other updates.

In response to the high level comments, which we understand to primarily relate to a) the improvements of GRCh38 over GRCh37 and b) comparison between *de novo* calling versus lift-over:

a) It was not our intention to demonstrate the superiority of GRCh38 over GRCh37. We believe that the GRC, in particular in the paper by Schneider *et al.*, have demonstrated this already. We included information on this for the information of readers who may not be familiar with these issues. However, we accept that this may give an inaccurate impression of the emphasis of the data note. As such, the text has been amended, reducing the explanation of assembly changes and, rather, making reference to the paper by Schnieder *et al*. Our aim was to provide a resource for those wishing to adopt the new assembly, not to present the case as to why GRCh38 should be adopted, which we believe has already been made elsewhere.

b) Our emphasis is on providing resources for the community. To make the data available to users in as timely a manner as possible, we elected to use the data note format for publication. This has a focus on describing how the data was produced, with validation of data outputs being listed in the information to authors as optional. In light of the comments, we have performed a comparison with the lift over set and also looked specifically at regions of the assembly that were updated between the two assemblies. Further details are below.

**1) Explanation of why “lift-over” approaches have limitations:**

This relates to a set of three statements regarding the inadequacy of lift-overs.

For the first statement, the reviewer notes that the removal of sequence when moving from GRCh37 to GRCh38 and the gain of sequence are two separate cases and that these were conflated in the statement “they rely on an equivalent region existing in the new genome, so new sequence in the improved assembly is effectively excluded”. We accept that this combines multiple facets of changes between the two assemblies. The text for statement one has, therefore, been updated to focus on the central point that we sought to make: that a mapping between the assemblies is necessary before one can lift-over a given variant and that this is not always possible (for any one of a number of possible reasons). Further, we have added the number of records that could not be lifted over in the dbSNP/EVA processed files to give a concrete indication of the numbers of records where this occurs.

For the second statement, the point we wished to make was that, even when a variant can be lifted-over, it does not follow that the evidence that supported that call in the original assembly would also transfer to the new location. The text has been amended in an effort to make this clearer, also citing evidence from Schneider *et al.* in regard to alignments and the transition from GRCh37 to GRCh38.

For the third statement, it was noted that this was clear but that no evidence was provided to support the assertion. In light of the other changes, this text has been modified to focus on the case of new sequence being added to the assembly and pointing to specific examples shown as part of Figure 1, which illustrate the differences in the lift-over and *de novo* call sets at examples of clinically relevant loci, which were updated between the two assemblies

**2) The authors provide only biallelic SNPs:**

The requested comparison with the lift-over is addressed in the response to point seven. We have added the requested numbers relating to what fraction of SNVs are biallelic (99.6%) and the number of SNVs relative to other short variants. We have also taken the opportunity to update the call set to include biallelic INDELs, a category of variant that was not previously included. Multi-allelic calls remain absent from the set as SHAPEIT is unable to handle such calls and our pipelines would require further development. Our strategy has been to release calls as soon as possible and to revisit the data set adding additional classes of variant as practical. This was done with the aim of making data useful to many available and with the intention of revisiting the data set to extend it as useful.

**3) Page 3, Quality control of alignment files:**

All of the steps used are described in the data note, not just the differences. The text has been updated in an effort to make this clearer to readers.

**4) Variant discovery:**

Tools were chosen in consultation with members of the 1000 Genomes Project Consortium. While our aim was to recapitulate their GRCh37 analysis on the new assembly, this would not have been feasible given the large number of callers used in the original project, the concomitant compute and the relatively complex methods used in filtering and integrating call sets, which were both compute and labour intensive. This obliged us to look at using a reduced set of callers and a simplified methodology. We sought recommendations that took into account the performance of the callers on the 1000 Genomes phase three data, which unlike most other panels is a mix of low coverage and exome with significantly more geographic diversity. In addition, the performance of some callers on the data set rendered their use impractical.

The text has been updated to inform readers of the above.

**5) Variant filtering:**

This was intended to be an initial release of data, with the intention of revisiting and adding additional elements that require further processing. As the sex chromosomes required additional analysis, they were not included in this first release. Further, we believe that the data set is still beneficial to some users in their absence. We anticipate calls on the GRCh38 sex chromosomes being released in the future.

**6) Data set validation:**

We acknowledge that the GIAB NA12878 benchmark is imperfect. As the reviewer notes, it is a single sample and the differences in the versions of the reference genome used for alignment (with and without alternate loci) by us and GIAB would be expected to have a bearing on variant detection.

With regard to the alternate loci, the possibility of comparing the level of conflict with the benchmark at regions where alternate loci are present and where they are not is mentioned. Given, however, that the presence of the alternate loci would also be expected to have at least some impact across the genome (irrespective of the presence of alternate loci at that particular location), we feel that, in order to truly assess what impact the alternate loci had on the analysis, it would be necessary to repeat the analysis, using, instead, alignments where the alternate loci had not been present. As our data set also relies on joint genotyping, this would effectively mean realigning all of the data and repeating analysis on all of the data in order to answer this question. The substantial compute volume involved in this would add significant time and expense and, thus, makes this comparison impractical. It would, however, be necessary in order to derive meaningful and sound conclusions about the impact of the alternate loci on our analysis.

The reviewer also expresses concern that accuracy with NA12878 may not transfer to other samples, particularly those of non-European ancestry. Given the prevalence of data from NA12878, it would seem reasonable to conclude that calling methods should perform well, and potentially above average, with that sample. However, NA12878 has data similar to that of other samples in our data set. Further, our data set is comprised only of Illumina data, so we do not expect, for example, types of sequencing error to vary across the samples. In work done by others, comparing the new call set to 1000 Genomes phase three, we see that our results and those for phase three show a strong level of consistency across the samples (Robinson and Glusman, 2019, https://www.biorxiv.org/content/10.1101/600254v1), with no indication that NA12878 is an outlier.

In relation to our comparison with phase three, it was not our intention to try to outperform phase three, rather to offer a *de novo* call set of similar quality on GRCh38. The utility is to those wishing to work with GRCh38 and work with a *de novo* call set generated on that assembly including the novel GRCh38 regions. The comparison with phase three is offered to assist users in understanding how our call set compares to phase three. Our call set shows broadly similar behaviour to phase three, with a slightly different balance of sensitivity and specificity. Given, however, that phase three involved a massively greater analysis effort, which resources would make impossible to repeat, it is perhaps not surprising that phase three sees a higher yield. In turn, this is reflected in the lift-over but with substantial differences demonstrated in novel regions where the *de novo* call set detects variants absent in the lift-over.

While we do acknowledge the limitations of the GIAB benchmark we have used, we found no better alternatives. To effectively benchmark our data, based as it is on joint genotyping, we needed “gold standard” data for samples in our data set. For short variants, the only such data set we were able to locate was GIAB NA12878. The alternatives, of manual inspection of the data or alternative data types, such as PacBio reads being assessed by us also have limitations and lose the benefits gained from an independent “gold standard” data set created by another group.

The text has been updated in an effort to better reflect the above.

**7) Omitted analysis:**

The alternate loci were used in aligning reads to ensure the best possible read mapping but variants were not called on these loci. The text has been amended to make this clearer. This is in large part as protocols for successfully calling on the alternate loci are lacking. The only information provided by developers of calling software of which we are aware in relation to this is a beta tutorial from GATK (https://software.broadinstitute.org/gatk/documentation/article.php?id=8017). Due to the lack of tools and protocols for confidently calling on the alternate loci, calls were not made at those loci.

We have extended our benchmarking work to include the lift-over data set in the comparison. We have also looked specifically at novel regions of GRCh38. These are included in the revised text.

The suggestion relating to fosmid clones is interesting and would provide further validation. We would, however, note that this is a data note, offered by the journal as a means of describing the production of a data set, where benchmarking is described as optional. Our existing benchmarking covers the genome at greater scale and should, therefore, already give a better indication of the performance of our calling genome wide. Further, we have added benchmarking of the phasing using WhatsHap.
